# Supplementary figures and images for: Small molecule inhibition of lysine-specific demethylase 1 (LSD1) and histone deacetylase (HDAC) alone and in combination in Ewing sarcoma cell lines
Source: PLoS One. 2019 Sep 24;14(9):e0222228. doi: 10.1371/journal.pone.0222228 (PMC6759167; doi:10.1371/journal.pone.0222228)

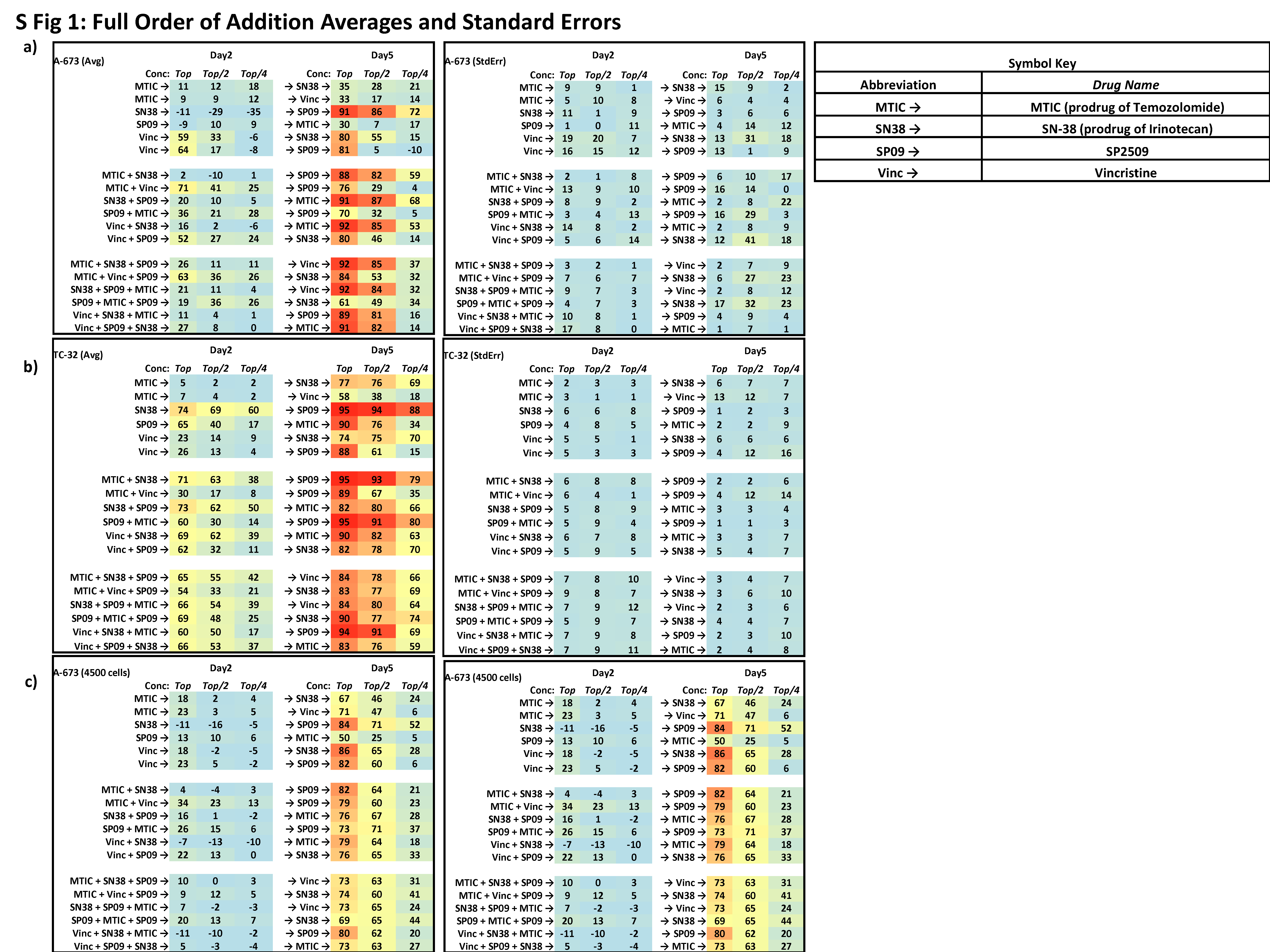

Supplement: S1 Fig — a) A673 (2250 cells), b) TC32 (4500 cells), c) A673 (4500 cells). (TIF) [file pone.0222228.s001.tif]

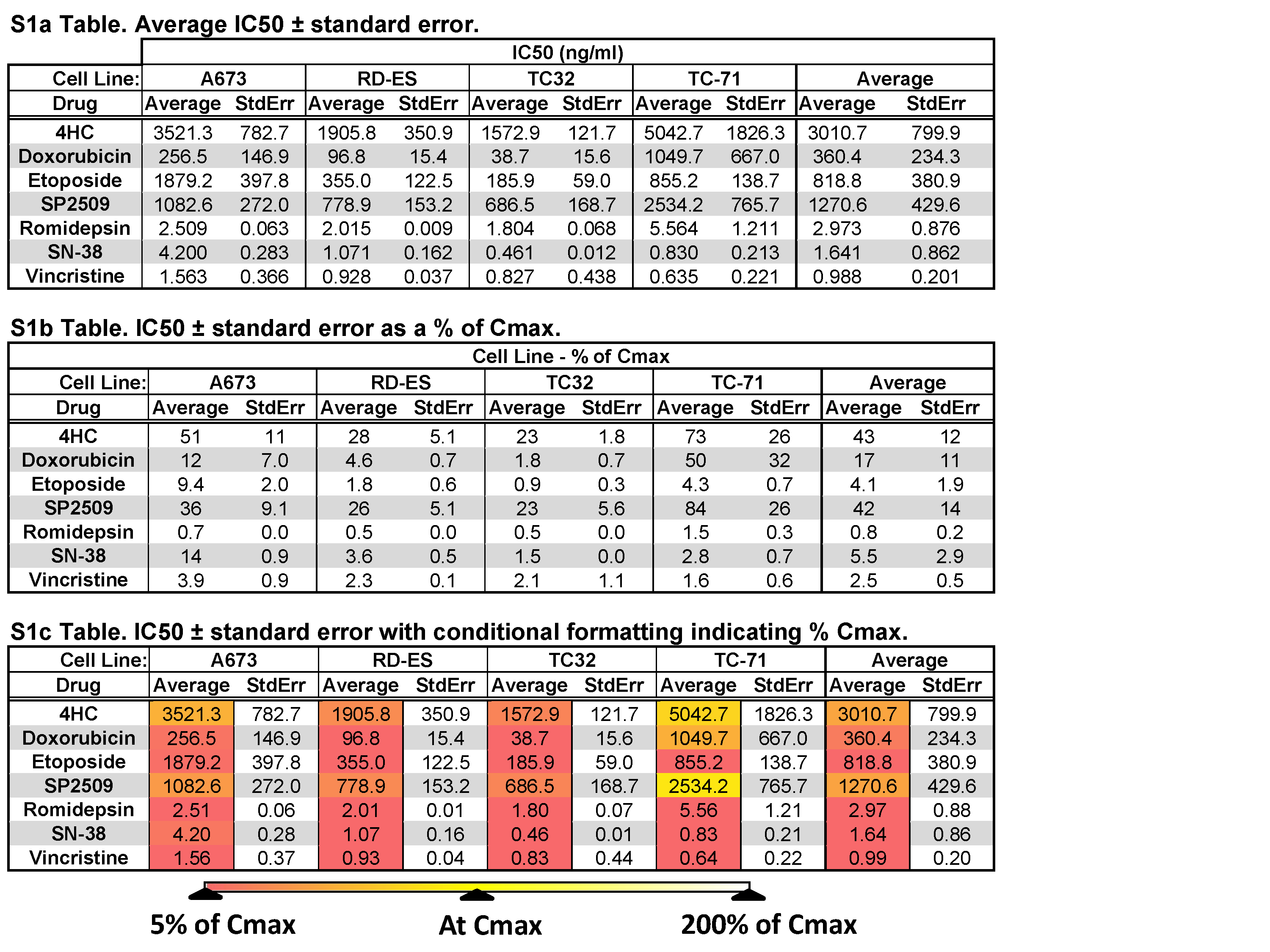

Supplement: S1 Table — A) Average IC50 ± standard error. B) IC50 ± standard error as a % of Cmax. C) IC50 ± standard error with conditional formatting indicating % Cmax. (TIFF) [file pone.0222228.s002.tiff]

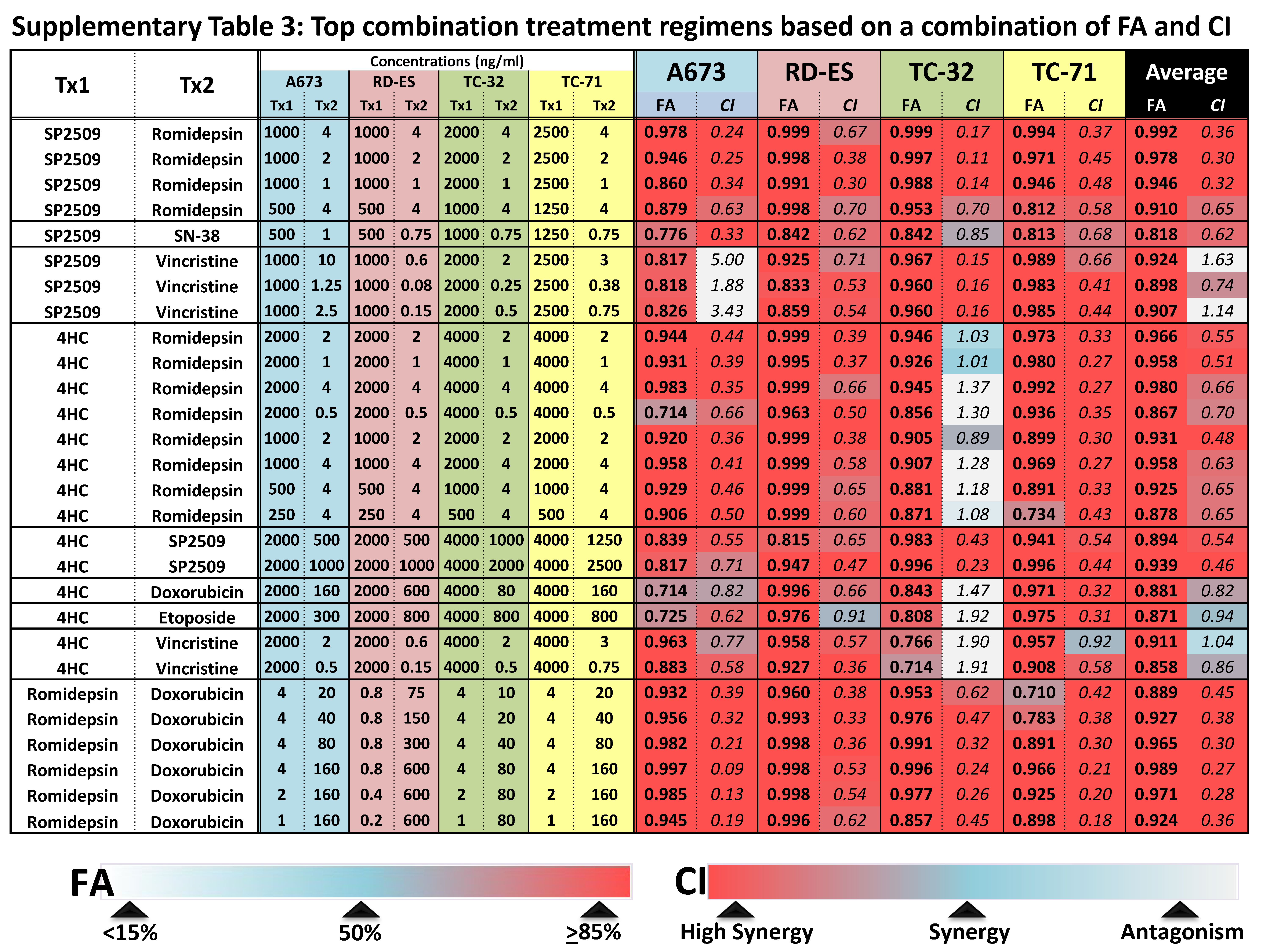

Supplement: S3 Table — (TIF) [file pone.0222228.s004.tif]
