# Supplementary material for: Small molecule inhibition of lysine-specific demethylase 1 (LSD1) and histone deacetylase (HDAC) alone and in combination in Ewing sarcoma cell lines
Source: PLoS One. 2019 Sep 24;14(9):e0222228. doi: 10.1371/journal.pone.0222228 (PMC6759167; doi:10.1371/journal.pone.0222228)
Supplement: S2 Table — (DOCX) [file pone.0222228.s003.docx]

**S2 Table. Full table of two-drug combinations**

| **Agents** | | **Concentrations (ng/ml)** | | | | | | | | **Fraction Affected (FA) and Combination Indices (CI)** | | | | | | | | | |
| --- | --- | --- | --- | --- | --- | --- | --- | --- | --- | --- | --- | --- | --- | --- | --- | --- | --- | --- | --- |
| **Tx1** | **Tx2** | **A673** | | **RD-ES** | | **TC32** | | **TC-71** | | **A673** | | **RD-ES** | | **TC32** | | **TC-71** | | **Average** | |
|  |  | **Tx1** | **Tx2** | **Tx1** | **Tx2** | **Tx1** | **Tx2** | **Tx1** | **Tx2** | **FA** | ***CI*** | **FA** | ***CI*** | **FA** | ***CI*** | **FA** | ***CI*** | **FA** | ***CI*** |
| **4HC** | **Doxorubicin** | 125 | 10 | 125 | 37.5 | 250 | 5 | 250 | 10 | **0.084** | *0.21* | **0.399** | *0.43* | **0.173** | *0.84* | **0.064** | *0.19* | **0.180** | *0.42* |
|  |  |  | 20 |  | 75 |  | 10 |  | 20 | **0.123** | *0.26* | **0.914** | *0.27* | **0.263** | *0.82* | **0.079** | *0.28* | **0.345** | *0.40* |
|  |  |  | 40 |  | 150 |  | 20 |  | 40 | **0.407** | *0.19* | **0.978** | *0.30* | **0.340** | *0.95* | **0.055** | *0.60* | **0.445** | *0.51* |
|  |  |  | 80 |  | 300 |  | 40 |  | 80 | **0.423** | *0.32* | **0.989** | *0.46* | **0.480** | *0.94* | **0.131** | *0.67* | **0.506** | *0.60* |
|  |  |  | 160 |  | 600 |  | 80 |  | 160 | **0.454** | *0.56* | **0.997** | *0.55* | **0.660** | *0.80* | **0.509** | *0.45* | **0.655** | *0.59* |
|  |  | 250 | 10 | 250 | 37.5 | 500 | 5 | 500 | 10 | **0.018** | *0.62* | **0.526** | *0.39* | **0.252** | *0.87* | **0.065** | *0.26* | **0.215** | *0.53* |
|  |  |  | 20 |  | 75 |  | 10 |  | 20 | **0.156** | *0.31* | **0.938** | *0.25* | **0.334** | *0.85* | **0.073** | *0.36* | **0.375** | *0.44* |
|  |  |  | 40 |  | 150 |  | 20 |  | 40 | **0.456** | *0.23* | **0.975** | *0.33* | **0.434** | *0.86* | **0.072** | *0.58* | **0.484** | *0.50* |
|  |  |  | 80 |  | 300 |  | 40 |  | 80 | **0.400** | *0.39* | **0.987** | *0.48* | **0.570** | *0.82* | **0.170** | *0.62* | **0.532** | *0.58* |
|  |  |  | 160 |  | 600 |  | 80 |  | 160 | **0.466** | *0.59* | **0.998** | *0.50* | **0.738** | *0.66* | **0.569** | *0.43* | **0.693** | *0.55* |
|  |  | 500 | 10 | 500 | 37.5 | 1000 | 5 | 1000 | 10 | **0.088** | *0.53* | **0.652** | *0.39* | **0.402** | *0.97* | **0.042** | *0.47* | **0.296** | *0.59* |
|  |  |  | 20 |  | 75 |  | 10 |  | 20 | **0.260** | *0.37* | **0.944** | *0.27* | **0.487** | *0.89* | **0.113** | *0.42* | **0.451** | *0.49* |
|  |  |  | 40 |  | 150 |  | 20 |  | 40 | **0.475** | *0.31* | **0.975** | *0.35* | **0.584** | *0.83* | **0.121** | *0.56* | **0.539** | *0.51* |
|  |  |  | 80 |  | 300 |  | 40 |  | 80 | **0.437** | *0.47* | **0.981** | *0.59* | **0.681** | *0.79* | **0.321** | *0.50* | **0.605** | *0.59* |
|  |  |  | 160 |  | 600 |  | 80 |  | 160 | **0.512** | *0.63* | **0.997** | *0.54* | **0.781** | *0.73* | **0.648** | *0.43* | **0.734** | *0.58* |
|  |  | 1000 | 10 | 1000 | 37.5 | 2000 | 5 | 2000 | 10 | **0.250** | *0.60* | **0.835** | *0.36* | **0.688** | *0.99* | **0.270** | *0.43* | **0.510** | *0.59* |
|  |  |  | 20 |  | 75 |  | 10 |  | 20 | **0.421** | *0.48* | **0.936** | *0.35* | **0.728** | *0.92* | **0.332** | *0.44* | **0.604** | *0.55* |
|  |  |  | 40 |  | 150 |  | 20 |  | 40 | **0.538** | *0.46* | **0.955** | *0.49* | **0.729** | *0.98* | **0.451** | *0.44* | **0.668** | *0.59* |
|  |  |  | 80 |  | 300 |  | 40 |  | 80 | **0.516** | *0.59* | **0.965** | *0.80* | **0.737** | *1.07* | **0.650** | *0.41* | **0.717** | *0.72* |
|  |  |  | 160 |  | 600 |  | 80 |  | 160 | **0.573** | *0.73* | **0.995** | *0.69* | **0.796** | *1.04* | **0.834** | *0.38* | **0.800** | *0.71* |
|  |  | 2000 | 10 | 2000 | 37.5 | 4000 | 5 | 4000 | 10 | **0.642** | *0.63* | **0.925** | *0.38* | **0.711** | *1.84* | **0.827** | *0.43* | **0.776** | *0.82* |
|  |  |  | 20 |  | 75 |  | 10 |  | 20 | **0.664** | *0.62* | **0.913** | *0.54* | **0.752** | *1.68* | **0.857** | *0.42* | **0.797** | *0.81* |
|  |  |  | 40 |  | 150 |  | 20 |  | 40 | **0.686** | *0.64* | **0.910** | *0.81* | **0.773** | *1.63* | **0.887** | *0.41* | **0.814** | *0.87* |
|  |  |  | 80 |  | 300 |  | 40 |  | 80 | **0.666** | *0.74* | **0.937** | *1.14* | **0.803** | *1.57* | **0.933** | *0.38* | **0.835** | *0.96* |
|  |  |  | 160 |  | 600 |  | 80 |  | 160 | **0.714** | *0.82* | **0.996** | *0.66* | **0.843** | *1.47* | **0.971** | *0.32* | **0.881** | *0.82* |
| **4HC** | **Etoposide** | 125 | 18.8 | 125 | 50 | 250 | 50 | 250 | 50 | **0.021** | *5.00* | **0.082** | *0.49* | **0.201** | *1.81* | **0.058** | *0.43* | **0.090** | *1.93* |
|  |  |  | 37.5 |  | 100 |  | 100 |  | 100 | **0.017** | *5.00* | **0.192** | *0.58* | **0.284** | *1.87* | **0.055** | *0.82* | **0.137** | *2.07* |
|  |  |  | 75 |  | 200 |  | 200 |  | 200 | **-0.016** | *5.00* | **0.589** | *0.56* | **0.354** | *2.30* | **0.091** | *1.07* | **0.254** | *2.23* |
|  |  |  | 150 |  | 400 |  | 400 |  | 400 | **-0.219** | *5.00* | **0.951** | *0.42* | **0.465** | *2.41* | **0.446** | *0.47* | **0.411** | *2.07* |
|  |  |  | 300 |  | 800 |  | 800 |  | 800 | **-0.058** | *5.00* | **0.991** | *0.44* | **0.676** | *1.48* | **0.724** | *0.39* | **0.583** | *1.83* |
|  |  | 250 | 18.8 | 250 | 50 | 500 | 50 | 500 | 50 | **0.073** | *1.33* | **0.129** | *0.54* | **0.243** | *1.66* | **0.063** | *0.48* | **0.127** | *1.00* |
|  |  |  | 37.5 |  | 100 |  | 100 |  | 100 | **0.035** | *5.00* | **0.256** | *0.61* | **0.320** | *1.77* | **0.056** | *0.89* | **0.167** | *2.07* |
|  |  |  | 75 |  | 200 |  | 200 |  | 200 | **0.017** | *5.00* | **0.613** | *0.59* | **0.419** | *1.83* | **0.127** | *0.88* | **0.294** | *2.08* |
|  |  |  | 150 |  | 400 |  | 400 |  | 400 | **-0.276** | *5.00* | **0.952** | *0.44* | **0.538** | *1.82* | **0.520** | *0.42* | **0.434** | *1.92* |
|  |  |  | 300 |  | 800 |  | 800 |  | 800 | **-0.034** | *5.00* | **0.991** | *0.45* | **0.716** | *1.28* | **0.731** | *0.41* | **0.601** | *1.79* |
|  |  | 500 | 18.8 | 500 | 50 | 1000 | 50 | 1000 | 50 | **0.040** | *4.45* | **0.265** | *0.59* | **0.338** | *1.53* | **0.058** | *0.66* | **0.175** | *1.81* |
|  |  |  | 37.5 |  | 100 |  | 100 |  | 100 | **0.009** | *5.00* | **0.365** | *0.67* | **0.426** | *1.49* | **0.101** | *0.72* | **0.225** | *1.97* |
|  |  |  | 75 |  | 200 |  | 200 |  | 200 | **-0.005** | *5.00* | **0.700** | *0.62* | **0.538** | *1.39* | **0.263** | *0.59* | **0.374** | *1.90* |
|  |  |  | 150 |  | 400 |  | 400 |  | 400 | **-0.271** | *5.00* | **0.964** | *0.44* | **0.681** | *1.14* | **0.625** | *0.39* | **0.500** | *1.74* |
|  |  |  | 300 |  | 800 |  | 800 |  | 800 | **0.069** | *1.02* | **0.988** | *0.52* | **0.769** | *1.12* | **0.768** | *0.42* | **0.649** | *0.77* |
|  |  | 1000 | 18.8 | 1000 | 50 | 2000 | 50 | 2000 | 50 | **0.091** | *1.53* | **0.621** | *0.57* | **0.588** | *1.31* | **0.319** | *0.45* | **0.405** | *0.96* |
|  |  |  | 37.5 |  | 100 |  | 100 |  | 100 | **0.065** | *3.75* | **0.739** | *0.57* | **0.674** | *1.15* | **0.457** | *0.42* | **0.484** | *1.47* |
|  |  |  | 75 |  | 200 |  | 200 |  | 200 | **0.077** | *4.79* | **0.890** | *0.53* | **0.745** | *1.05* | **0.618** | *0.40* | **0.582** | *1.69* |
|  |  |  | 150 |  | 400 |  | 400 |  | 400 | **0.012** | *2.55* | **0.978** | *0.44* | **0.781** | *1.09* | **0.809** | *0.34* | **0.645** | *1.10* |
|  |  |  | 300 |  | 800 |  | 800 |  | 800 | **0.361** | *0.63* | **0.986** | *0.63* | **0.780** | *1.42* | **0.879** | *0.36* | **0.751** | *0.76* |
|  |  | 2000 | 18.8 | 2000 | 50 | 4000 | 50 | 4000 | 50 | **0.524** | *0.72* | **0.955** | *0.42* | **0.677** | *2.03* | **0.874** | *0.40* | **0.758** | *0.89* |
|  |  |  | 37.5 |  | 100 |  | 100 |  | 100 | **0.524** | *0.73* | **0.962** | *0.44* | **0.711** | *1.94* | **0.893** | *0.39* | **0.773** | *0.88* |
|  |  |  | 75 |  | 200 |  | 200 |  | 200 | **0.527** | *0.74* | **0.971** | *0.48* | **0.766** | *1.76* | **0.923** | *0.37* | **0.797** | *0.84* |
|  |  |  | 150 |  | 400 |  | 400 |  | 400 | **0.595** | *0.72* | **0.975** | *0.61* | **0.793** | *1.76* | **0.949** | *0.35* | **0.828** | *0.86* |
|  |  |  | 300 |  | 800 |  | 800 |  | 800 | **0.725** | *0.62* | **0.976** | *0.91* | **0.808** | *1.92* | **0.975** | *0.31* | **0.871** | *0.94* |
| **4HC** | **Romidepsin** | 125 | 0.25 | 125 | 0.25 | 250 | 0.25 | 250 | 0.25 | **0.089** | *0.48* | **0.027** | *0.51* | **0.042** | *1.19* | **0.068** | *0.17* | **0.056** | *0.59* |
|  |  |  | 0.5 |  | 0.5 |  | 0.5 |  | 0.5 | **0.116** | *0.72* | **0.060** | *0.64* | **0.073** | *1.24* | **0.079** | *0.26* | **0.082** | *0.72* |
|  |  |  | 1 |  | 1 |  | 1 |  | 1 | **0.265** | *0.78* | **0.410** | *0.67* | **0.131** | *1.46* | **0.082** | *0.45* | **0.222** | *0.84* |
|  |  |  | 2 |  | 2 |  | 2 |  | 2 | **0.869** | *0.31* | **0.997** | *0.37* | **0.632** | *0.93* | **0.262** | *0.48* | **0.690** | *0.52* |
|  |  |  | 4 |  | 4 |  | 4 |  | 4 | **0.899** | *0.51* | **0.999** | *0.62* | **0.864** | *1.04* | **0.675** | *0.45* | **0.859** | *0.65* |
|  |  | 250 | 0.25 | 250 | 0.25 | 500 | 0.25 | 500 | 0.25 | **0.090** | *0.58* | **0.049** | *0.62* | **0.089** | *1.36* | **0.068** | *0.24* | **0.074** | *0.70* |
|  |  |  | 0.5 |  | 0.5 |  | 0.5 |  | 0.5 | **0.140** | *0.73* | **0.060** | *0.81* | **0.115** | *1.46* | **0.077** | *0.33* | **0.098** | *0.83* |
|  |  |  | 1 |  | 1 |  | 1 |  | 1 | **0.309** | *0.76* | **0.518** | *0.68* | **0.240** | *1.37* | **0.112** | *0.45* | **0.295** | *0.81* |
|  |  |  | 2 |  | 2 |  | 2 |  | 2 | **0.880** | *0.31* | **0.997** | *0.39* | **0.708** | *0.93* | **0.314** | *0.48* | **0.725** | *0.53* |
|  |  |  | 4 |  | 4 |  | 4 |  | 4 | **0.906** | *0.50* | **0.999** | *0.60* | **0.871** | *1.08* | **0.734** | *0.43* | **0.878** | *0.65* |
|  |  | 500 | 0.25 | 500 | 0.25 | 1000 | 0.25 | 1000 | 0.25 | **0.144** | *0.62* | **0.155** | *0.67* | **0.242** | *1.39* | **0.066** | *0.39* | **0.152** | *0.77* |
|  |  |  | 0.5 |  | 0.5 |  | 0.5 |  | 0.5 | **0.091** | *1.15* | **0.198** | *0.80* | **0.262** | *1.51* | **0.094** | *0.44* | **0.161** | *0.97* |
|  |  |  | 1 |  | 1 |  | 1 |  | 1 | **0.440** | *0.67* | **0.761** | *0.63* | **0.459** | *1.28* | **0.128** | *0.55* | **0.447** | *0.78* |
|  |  |  | 2 |  | 2 |  | 2 |  | 2 | **0.897** | *0.33* | **0.998** | *0.37* | **0.829** | *0.86* | **0.536** | *0.41* | **0.815** | *0.49* |
|  |  |  | 4 |  | 4 |  | 4 |  | 4 | **0.929** | *0.46* | **0.999** | *0.65* | **0.881** | *1.18* | **0.891** | *0.33* | **0.925** | *0.65* |
|  |  | 1000 | 0.25 | 1000 | 0.25 | 2000 | 0.25 | 2000 | 0.25 | **0.150** | *0.96* | **0.533** | *0.65* | **0.579** | *1.30* | **0.293** | *0.43* | **0.389** | *0.83* |
|  |  |  | 0.5 |  | 0.5 |  | 0.5 |  | 0.5 | **0.214** | *1.01* | **0.608** | *0.72* | **0.687** | *1.13* | **0.354** | *0.44* | **0.466** | *0.82* |
|  |  |  | 1 |  | 1 |  | 1 |  | 1 | **0.750** | *0.47* | **0.978** | *0.42* | **0.882** | *0.76* | **0.525** | *0.43* | **0.784** | *0.52* |
|  |  |  | 2 |  | 2 |  | 2 |  | 2 | **0.920** | *0.36* | **0.999** | *0.38* | **0.905** | *0.89* | **0.899** | *0.30* | **0.931** | *0.48* |
|  |  |  | 4 |  | 4 |  | 4 |  | 4 | **0.958** | *0.41* | **0.999** | *0.58* | **0.907** | *1.28* | **0.969** | *0.27* | **0.958** | *0.63* |
|  |  | 2000 | 0.25 | 2000 | 0.25 | 4000 | 0.25 | 4000 | 0.25 | **0.604** | *0.72* | **0.930** | *0.53* | **0.724** | *1.83* | **0.885** | *0.39* | **0.786** | *0.87* |
|  |  |  | 0.5 |  | 0.5 |  | 0.5 |  | 0.5 | **0.714** | *0.66* | **0.963** | *0.50* | **0.856** | *1.30* | **0.936** | *0.35* | **0.867** | *0.70* |
|  |  |  | 1 |  | 1 |  | 1 |  | 1 | **0.931** | *0.39* | **0.995** | *0.37* | **0.926** | *1.01* | **0.980** | *0.27* | **0.958** | *0.51* |
|  |  |  | 2 |  | 2 |  | 2 |  | 2 | **0.944** | *0.44* | **0.999** | *0.39* | **0.946** | *1.03* | **0.973** | *0.33* | **0.966** | *0.55* |
|  |  |  | 4 |  | 4 |  | 4 |  | 4 | **0.983** | *0.35* | **0.999** | *0.66* | **0.945** | *1.37* | **0.992** | *0.27* | **0.980** | *0.66* |
| **4HC** | **SN-38** | 125 | 0.0625 | 125 | 0.047 | 250 | 0.047 | 250 | 0.047 | **0.056** | *0.16* | **0.021** | *0.35* | **0.095** | *0.93* | **0.022** | *0.21* | **0.049** | *0.41* |
|  |  |  | 0.125 |  | 0.094 |  | 0.094 |  | 0.094 | **0.050** | *0.20* | **0.019** | *0.45* | **0.125** | *1.13* | **0.035** | *0.29* | **0.057** | *0.52* |
|  |  |  | 0.25 |  | 0.188 |  | 0.188 |  | 0.188 | **0.011** | *0.44* | **0.048** | *0.49* | **0.199** | *1.31* | **0.039** | *0.47* | **0.074** | *0.68* |
|  |  |  | 0.5 |  | 0.375 |  | 0.375 |  | 0.375 | **0.030** | *0.48* | **0.165** | *0.57* | **0.400** | *1.25* | **0.354** | *0.41* | **0.237** | *0.68* |
|  |  |  | 1 |  | 0.75 |  | 0.75 |  | 0.75 | **0.161** | *0.48* | **0.761** | *0.54* | **0.808** | *0.74* | **0.737** | *0.49* | **0.617** | *0.56* |
|  |  | 250 | 0.0625 | 250 | 0.047 | 500 | 0.047 | 500 | 0.047 | **0.084** | *0.25* | **0.037** | *0.50* | **0.183** | *0.97* | **0.037** | *0.26* | **0.085** | *0.49* |
|  |  |  | 0.125 |  | 0.094 |  | 0.094 |  | 0.094 | **0.028** | *0.41* | **0.055** | *0.51* | **0.211** | *1.12* | **0.065** | *0.31* | **0.090** | *0.59* |
|  |  |  | 0.25 |  | 0.188 |  | 0.188 |  | 0.188 | **0.046** | *0.42* | **0.087** | *0.57* | **0.290** | *1.24* | **0.085** | *0.44* | **0.127** | *0.67* |
|  |  |  | 0.5 |  | 0.375 |  | 0.375 |  | 0.375 | **0.046** | *0.56* | **0.246** | *0.60* | **0.531** | *1.08* | **0.437** | *0.41* | **0.315** | *0.66* |
|  |  |  | 1 |  | 0.75 |  | 0.75 |  | 0.75 | **0.224** | *0.50* | **0.827** | *0.53* | **0.836** | *0.74* | **0.756** | *0.50* | **0.661** | *0.57* |
|  |  | 500 | 0.0625 | 500 | 0.047 | 1000 | 0.047 | 1000 | 0.047 | **0.051** | *0.56* | **0.156** | *0.54* | **0.347** | *1.09* | **0.062** | *0.38* | **0.154** | *0.64* |
|  |  |  | 0.125 |  | 0.094 |  | 0.094 |  | 0.094 | **0.048** | *0.61* | **0.191** | *0.55* | **0.392** | *1.12* | **0.091** | *0.41* | **0.180** | *0.67* |
|  |  |  | 0.25 |  | 0.188 |  | 0.188 |  | 0.188 | **0.053** | *0.66* | **0.250** | *0.60* | **0.523** | *1.05* | **0.255** | *0.41* | **0.271** | *0.68* |
|  |  |  | 0.5 |  | 0.375 |  | 0.375 |  | 0.375 | **0.121** | *0.59* | **0.443** | *0.62* | **0.778** | *0.75* | **0.585** | *0.42* | **0.481** | *0.60* |
|  |  |  | 1 |  | 0.75 |  | 0.75 |  | 0.75 | **0.382** | *0.51* | **0.915** | *0.50* | **0.866** | *0.78* | **0.832** | *0.49* | **0.749** | *0.57* |
|  |  | 1000 | 0.0625 | 1000 | 0.047 | 2000 | 0.047 | 2000 | 0.047 | **0.174** | *0.67* | **0.561** | *0.53* | **0.665** | *1.06* | **0.352** | *0.40* | **0.438** | *0.67* |
|  |  |  | 0.125 |  | 0.094 |  | 0.094 |  | 0.094 | **0.213** | *0.64* | **0.622** | *0.52* | **0.734** | *0.96* | **0.480** | *0.39* | **0.512** | *0.63* |
|  |  |  | 0.25 |  | 0.188 |  | 0.188 |  | 0.188 | **0.273** | *0.61* | **0.722** | *0.52* | **0.831** | *0.80* | **0.638** | *0.40* | **0.616** | *0.58* |
|  |  |  | 0.5 |  | 0.375 |  | 0.375 |  | 0.375 | **0.395** | *0.57* | **0.877** | *0.48* | **0.900** | *0.69* | **0.781** | *0.44* | **0.738** | *0.54* |
|  |  |  | 1 |  | 0.75 |  | 0.75 |  | 0.75 | **0.543** | *0.59* | **0.974** | *0.44* | **0.915** | *0.81* | **0.905** | *0.50* | **0.834** | *0.58* |
|  |  | 2000 | 0.0625 | 2000 | 0.047 | 4000 | 0.047 | 4000 | 0.047 | **0.583** | *0.67* | **0.959** | *0.38* | **0.638** | *2.18* | **0.874** | *0.41* | **0.764** | *0.91* |
|  |  |  | 0.125 |  | 0.094 |  | 0.094 |  | 0.094 | **0.613** | *0.66* | **0.964** | *0.38* | **0.649** | *2.20* | **0.904** | *0.40* | **0.783** | *0.91* |
|  |  |  | 0.25 |  | 0.188 |  | 0.188 |  | 0.188 | **0.635** | *0.66* | **0.969** | *0.40* | **0.645** | *2.36* | **0.927** | *0.41* | **0.794** | *0.96* |
|  |  |  | 0.5 |  | 0.375 |  | 0.375 |  | 0.375 | **0.679** | *0.67* | **0.974** | *0.45* | **0.658** | *2.56* | **0.954** | *0.43* | **0.816** | *1.03* |
|  |  |  | 1 |  | 0.75 |  | 0.75 |  | 0.75 | **0.703** | *0.75* | **0.977** | *0.58* | **0.680** | *2.94* | **0.978** | *0.45* | **0.834** | *1.18* |
| **4HC** | **SP2509** | 125 | 62.5 | 125 | 62.5 | 250 | 125 | 250 | 157 | **0.000** | *5.00* | **-0.009** | *5.00* | **0.058** | *2.52* | **0.039** | *0.16* | **0.022** | *3.17* |
|  |  |  | 125 |  | 125 |  | 250 |  | 313 | **-0.024** | *5.00* | **0.026** | *1.07* | **0.170** | *1.65* | **0.016** | *0.28* | **0.047** | *2.00* |
|  |  |  | 250 |  | 250 |  | 500 |  | 625 | **0.283** | *0.50* | **0.223** | *0.59* | **0.455** | *0.93* | **0.019** | *0.44* | **0.245** | *0.61* |
|  |  |  | 500 |  | 500 |  | 1000 |  | 1250 | **0.465** | *0.51* | **0.520** | *0.56* | **0.555** | *1.19* | **0.061** | *0.63* | **0.400** | *0.72* |
|  |  |  | 1000 |  | 1000 |  | 2000 |  | 2500 | **0.455** | *1.00* | **0.888** | *0.39* | **0.956** | *0.21* | **0.983** | *0.37* | **0.820** | *0.49* |
|  |  | 250 | 62.5 | 250 | 62.5 | 500 | 125 | 500 | 157 | **-0.014** | *5.00* | **0.001** | *4.06* | **0.122** | *1.80* | **0.019** | *0.28* | **0.032** | *2.78* |
|  |  |  | 125 |  | 125 |  | 250 |  | 313 | **-0.002** | *5.00* | **0.041** | *1.00* | **0.245** | *1.45* | **0.011** | *0.41* | **0.074** | *1.96* |
|  |  |  | 250 |  | 250 |  | 500 |  | 625 | **0.295** | *0.54* | **0.254** | *0.61* | **0.644** | *0.63* | **0.029** | *0.49* | **0.306** | *0.57* |
|  |  |  | 500 |  | 500 |  | 1000 |  | 1250 | **0.483** | *0.53* | **0.591** | *0.52* | **0.650** | *0.99* | **0.047** | *0.74* | **0.443** | *0.70* |
|  |  |  | 1000 |  | 1000 |  | 2000 |  | 2500 | **0.494** | *0.93* | **0.907** | *0.37* | **0.963** | *0.22* | **0.987** | *0.37* | **0.838** | *0.47* |
|  |  | 500 | 62.5 | 500 | 62.5 | 1000 | 125 | 1000 | 157 | **-0.022** | *5.00* | **0.048** | *0.90* | **0.297** | *1.39* | **-0.006** | *5.00* | **0.079** | *3.07* |
|  |  |  | 125 |  | 125 |  | 250 |  | 313 | **0.046** | *1.70* | **0.140** | *0.71* | **0.571** | *0.85* | **0.033** | *0.50* | **0.197** | *0.94* |
|  |  |  | 250 |  | 250 |  | 500 |  | 625 | **0.382** | *0.52* | **0.422** | *0.52* | **0.816** | *0.51* | **0.026** | *0.69* | **0.412** | *0.56* |
|  |  |  | 500 |  | 500 |  | 1000 |  | 1250 | **0.570** | *0.50* | **0.634** | *0.55* | **0.711** | *1.02* | **0.212** | *0.65* | **0.532** | *0.68* |
|  |  |  | 1000 |  | 1000 |  | 2000 |  | 2500 | **0.556** | *0.86* | **0.928** | *0.35* | **0.966** | *0.27* | **0.990** | *0.37* | **0.860** | *0.46* |
|  |  | 1000 | 62.5 | 1000 | 62.5 | 2000 | 125 | 2000 | 157 | **0.142** | *0.92* | **0.407** | *0.50* | **0.588** | *1.28* | **0.166** | *0.51* | **0.326** | *0.80* |
|  |  |  | 125 |  | 125 |  | 250 |  | 313 | **0.367** | *0.61* | **0.542** | *0.46* | **0.752** | *0.94* | **0.243** | *0.51* | **0.476** | *0.63* |
|  |  |  | 250 |  | 250 |  | 500 |  | 625 | **0.517** | *0.56* | **0.758** | *0.38* | **0.776** | *0.98* | **0.491** | *0.49* | **0.635** | *0.60* |
|  |  |  | 500 |  | 500 |  | 1000 |  | 1250 | **0.676** | *0.51* | **0.744** | *0.54* | **0.853** | *0.87* | **0.550** | *0.63* | **0.706** | *0.64* |
|  |  |  | 1000 |  | 1000 |  | 2000 |  | 2500 | **0.618** | *0.87* | **0.936** | *0.39* | **0.977** | *0.32* | **0.994** | *0.39* | **0.881** | *0.50* |
|  |  | 2000 | 62.5 | 2000 | 62.5 | 4000 | 125 | 4000 | 157 | **0.574** | *0.71* | **0.857** | *0.38* | **0.666** | *2.08* | **0.810** | *0.47* | **0.727** | *0.91* |
|  |  |  | 125 |  | 125 |  | 250 |  | 313 | **0.643** | *0.68* | **0.870** | *0.39* | **0.702** | *1.99* | **0.840** | *0.48* | **0.763** | *0.88* |
|  |  |  | 250 |  | 250 |  | 500 |  | 625 | **0.700** | *0.67* | **0.870** | *0.43* | **0.800** | *1.61* | **0.871** | *0.52* | **0.810** | *0.81* |
|  |  |  | 500 |  | 500 |  | 1000 |  | 1250 | **0.839** | *0.55* | **0.815** | *0.65* | **0.983** | *0.43* | **0.941** | *0.54* | **0.894** | *0.54* |
|  |  |  | 1000 |  | 1000 |  | 2000 |  | 2500 | **0.817** | *0.71* | **0.947** | *0.47* | **0.996** | *0.23* | **0.996** | *0.44* | **0.939** | *0.46* |
| **4HC** | **Vincristine** | 125 | 0.125 | 125 | 0.0375 | 250 | 0.125 | 250 | 0.188 | **0.121** | *0.27* | **0.006** | *0.44* | **0.026** | *5.00* | **0.060** | *0.66* | **0.053** | *1.59* |
|  |  |  | 0.25 |  | 0.075 |  | 0.25 |  | 0.375 | **-0.096** | *5.00* | **0.001** | *0.95* | **0.062** | *5.00* | **0.126** | *0.86* | **0.023** | *2.95* |
|  |  |  | 0.5 |  | 0.15 |  | 0.5 |  | 0.75 | **0.914** | *0.20* | **0.007** | *0.78* | **0.214** | *2.56* | **0.577** | *0.60* | **0.428** | *1.04* |
|  |  |  | 1 |  | 0.3 |  | 1 |  | 1.5 | **0.963** | *0.28* | **0.025** | *0.84* | **0.371** | *1.82* | **0.900** | *0.50* | **0.565** | *0.86* |
|  |  |  | 2 |  | 0.6 |  | 2 |  | 3 | **0.973** | *0.48* | **0.523** | *0.69* | **0.574** | *1.22* | **0.973** | *0.52* | **0.761** | *0.73* |
|  |  | 250 | 0.125 | 250 | 0.0375 | 500 | 0.125 | 500 | 0.188 | **0.183** | *0.31* | **0.012** | *0.61* | **0.078** | *3.78* | **0.052** | *0.78* | **0.081** | *1.37* |
|  |  |  | 0.25 |  | 0.075 |  | 0.25 |  | 0.375 | **-0.153** | *5.00* | **0.014** | *0.78* | **0.131** | *3.29* | **0.144** | *0.86* | **0.034** | *2.48* |
|  |  |  | 0.5 |  | 0.15 |  | 0.5 |  | 0.75 | **0.910** | *0.22* | **0.023** | *0.82* | **0.270** | *2.04* | **0.607** | *0.60* | **0.453** | *0.92* |
|  |  |  | 1 |  | 0.3 |  | 1 |  | 1.5 | **0.966** | *0.28* | **0.030** | *0.98* | **0.424** | *1.60* | **0.882** | *0.57* | **0.575** | *0.86* |
|  |  |  | 2 |  | 0.6 |  | 2 |  | 3 | **0.975** | *0.49* | **0.469** | *0.77* | **0.636** | *1.02* | **0.959** | *0.64* | **0.760** | *0.73* |
|  |  | 500 | 0.125 | 500 | 0.0375 | 1000 | 0.125 | 1000 | 0.188 | **0.100** | *0.59* | **0.101** | *0.49* | **0.234** | *1.70* | **0.056** | *0.90* | **0.123** | *0.92* |
|  |  |  | 0.25 |  | 0.075 |  | 0.25 |  | 0.375 | **-0.207** | *5.00* | **0.091** | *0.57* | **0.287** | *1.74* | **0.206** | *0.82* | **0.094** | *2.03* |
|  |  |  | 0.5 |  | 0.15 |  | 0.5 |  | 0.75 | **0.904** | *0.27* | **0.124** | *0.62* | **0.389** | *1.58* | **0.659** | *0.61* | **0.519** | *0.77* |
|  |  |  | 1 |  | 0.3 |  | 1 |  | 1.5 | **0.974** | *0.29* | **0.154** | *0.80* | **0.560** | *1.20* | **0.856** | *0.67* | **0.636** | *0.74* |
|  |  |  | 2 |  | 0.6 |  | 2 |  | 3 | **0.979** | *0.48* | **0.661** | *0.72* | **0.677** | *1.08* | **0.949** | *0.75* | **0.817** | *0.76* |
|  |  | 1000 | 0.125 | 1000 | 0.0375 | 2000 | 0.125 | 2000 | 0.188 | **-0.036** | *5.00* | **0.495** | *0.40* | **0.568** | *1.29* | **0.257** | *0.66* | **0.321** | *1.84* |
|  |  |  | 0.25 |  | 0.075 |  | 0.25 |  | 0.375 | **-0.139** | *5.00* | **0.556** | *0.40* | **0.632** | *1.17* | **0.494** | *0.64* | **0.386** | *1.80* |
|  |  |  | 0.5 |  | 0.15 |  | 0.5 |  | 0.75 | **0.904** | *0.36* | **0.599** | *0.46* | **0.716** | *1.01* | **0.738** | *0.64* | **0.739** | *0.62* |
|  |  |  | 1 |  | 0.3 |  | 1 |  | 1.5 | **0.976** | *0.33* | **0.675** | *0.55* | **0.780** | *0.90* | **0.854** | *0.78* | **0.821** | *0.64* |
|  |  |  | 2 |  | 0.6 |  | 2 |  | 3 | **0.980** | *0.53* | **0.910** | *0.57* | **0.814** | *0.91* | **0.920** | *1.02* | **0.906** | *0.76* |
|  |  | 2000 | 0.125 | 2000 | 0.0375 | 4000 | 0.125 | 4000 | 0.188 | **0.190** | *1.40* | **0.909** | *0.31* | **0.682** | *1.96* | **0.830** | *0.50* | **0.653** | *1.04* |
|  |  |  | 0.25 |  | 0.075 |  | 0.25 |  | 0.375 | **0.406** | *1.08* | **0.921** | *0.32* | **0.694** | *1.94* | **0.871** | *0.52* | **0.723** | *0.97* |
|  |  |  | 0.5 |  | 0.15 |  | 0.5 |  | 0.75 | **0.883** | *0.58* | **0.927** | *0.36* | **0.714** | *1.91* | **0.908** | *0.58* | **0.858** | *0.86* |
|  |  |  | 1 |  | 0.3 |  | 1 |  | 1.5 | **0.953** | *0.56* | **0.930** | *0.46* | **0.721** | *1.99* | **0.932** | *0.72* | **0.884** | *0.93* |
|  |  |  | 2 |  | 0.6 |  | 2 |  | 3 | **0.963** | *0.77* | **0.958** | *0.57* | **0.766** | *1.90* | **0.957** | *0.92* | **0.911** | *1.04* |
| **Etoposide** | **Doxorubicin** | 18.8 | 10 | 18.8 | 37.5 | 50 | 5 | 50 | 10 | **0.051** | *2.47* | **0.231** | *0.56* | **0.248** | *1.32* | **0.004** | *3.09* | **0.133** | *1.86* |
|  |  |  | 20 |  | 75 |  | 10 |  | 20 | **0.107** | *0.65* | **0.859** | *0.33* | **0.318** | *1.04* | **0.058** | *0.61* | **0.336** | *0.66* |
|  |  |  | 40 |  | 150 |  | 20 |  | 40 | **0.389** | *0.16* | **0.969** | *0.34* | **0.392** | *0.97* | **0.030** | *1.33* | **0.445** | *0.70* |
|  |  |  | 80 |  | 300 |  | 40 |  | 80 | **0.451** | *0.27* | **0.988** | *0.46* | **0.509** | *0.89* | **0.072** | *1.19* | **0.505** | *0.70* |
|  |  |  | 160 |  | 600 |  | 80 |  | 160 | **0.427** | *0.55* | **0.995** | *0.67* | **0.651** | *0.80* | **0.432** | *0.55* | **0.626** | *0.64* |
|  |  | 37.5 | 10 | 37.5 | 37.5 | 100 | 5 | 100 | 10 | **0.024** | *5.00* | **0.338** | *0.50* | **0.357** | *1.18* | **0.038** | *1.17* | **0.189** | *1.96* |
|  |  |  | 20 |  | 75 |  | 10 |  | 20 | **0.151** | *0.58* | **0.898** | *0.30* | **0.398** | *1.07* | **0.062** | *0.92* | **0.377** | *0.72* |
|  |  |  | 40 |  | 150 |  | 20 |  | 40 | **0.446** | *0.15* | **0.974** | *0.33* | **0.453** | *1.02* | **0.054** | *1.28* | **0.482** | *0.69* |
|  |  |  | 80 |  | 300 |  | 40 |  | 80 | **0.438** | *0.29* | **0.989** | *0.45* | **0.539** | *0.96* | **0.128** | *1.00* | **0.524** | *0.67* |
|  |  |  | 160 |  | 600 |  | 80 |  | 160 | **0.459** | *0.52* | **0.997** | *0.55* | **0.672** | *0.81* | **0.446** | *0.58* | **0.643** | *0.62* |
|  |  | 75 | 10 | 75 | 37.5 | 200 | 5 | 200 | 10 | **0.061** | *5.00* | **0.454** | *0.49* | **0.435** | *1.42* | **0.073** | *1.31* | **0.256** | *2.05* |
|  |  |  | 20 |  | 75 |  | 10 |  | 20 | **0.182** | *0.69* | **0.922** | *0.29* | **0.460** | *1.35* | **0.057** | *1.72* | **0.405** | *1.01* |
|  |  |  | 40 |  | 150 |  | 20 |  | 40 | **0.475** | *0.16* | **0.979** | *0.31* | **0.490** | *1.33* | **0.130** | *1.05* | **0.518** | *0.71* |
|  |  |  | 80 |  | 300 |  | 40 |  | 80 | **0.451** | *0.30* | **0.989** | *0.46* | **0.576** | *1.11* | **0.278** | *0.73* | **0.573** | *0.65* |
|  |  |  | 160 |  | 600 |  | 80 |  | 160 | **0.488** | *0.51* | **0.996** | *0.58* | **0.698** | *0.86* | **0.546** | *0.54* | **0.682** | *0.62* |
|  |  | 150 | 10 | 150 | 37.5 | 400 | 5 | 400 | 10 | **0.068** | *5.00* | **0.646** | *0.46* | **0.513** | *1.79* | **0.146** | *1.42* | **0.343** | *2.17* |
|  |  |  | 20 |  | 75 |  | 10 |  | 20 | **0.237** | *0.69* | **0.942** | *0.29* | **0.519** | *1.82* | **0.240** | *0.96* | **0.484** | *0.94* |
|  |  |  | 40 |  | 150 |  | 20 |  | 40 | **0.507** | *0.17* | **0.979** | *0.34* | **0.560** | *1.59* | **0.356** | *0.71* | **0.600** | *0.70* |
|  |  |  | 80 |  | 300 |  | 40 |  | 80 | **0.482** | *0.31* | **0.990** | *0.47* | **0.626** | *1.31* | **0.552** | *0.50* | **0.662** | *0.65* |
|  |  |  | 160 |  | 600 |  | 80 |  | 160 | **0.513** | *0.50* | **0.997** | *0.58* | **0.723** | *0.99* | **0.684** | *0.48* | **0.729** | *0.64* |
|  |  | 300 | 10 | 300 | 37.5 | 800 | 5 | 800 | 10 | **0.165** | *2.92* | **0.855** | *0.40* | **0.673** | *1.43* | **0.639** | *0.50* | **0.583** | *1.31* |
|  |  |  | 20 |  | 75 |  | 10 |  | 20 | **0.337** | *0.53* | **0.964** | *0.30* | **0.685** | *1.37* | **0.641** | *0.51* | **0.657** | *0.68* |
|  |  |  | 40 |  | 150 |  | 20 |  | 40 | **0.556** | *0.18* | **0.982** | *0.36* | **0.697** | *1.34* | **0.652** | *0.53* | **0.722** | *0.60* |
|  |  |  | 80 |  | 300 |  | 40 |  | 80 | **0.530** | *0.31* | **0.989** | *0.52* | **0.725** | *1.24* | **0.697** | *0.53* | **0.735** | *0.65* |
|  |  |  | 160 |  | 600 |  | 80 |  | 160 | **0.540** | *0.51* | **0.994** | *0.75* | **0.765** | *1.14* | **0.789** | *0.48* | **0.772** | *0.72* |
| **Etoposide** | **Romidepsin** | 18.8 | 0.25 | 18.8 | 0.05 | 50 | 0.25 | 50 | 0.25 | **0.058** | *2.27* | **0.028** | *0.20* | **0.275** | *1.03* | **0.062** | *0.45* | **0.106** | *0.99* |
|  |  |  | 0.5 |  | 0.1 |  | 0.5 |  | 0.5 | **0.109** | *1.10* | **0.007** | *0.41* | **0.286** | *1.14* | **0.061** | *0.56* | **0.116** | *0.80* |
|  |  |  | 1 |  | 0.2 |  | 1 |  | 1 | **0.304** | *0.68* | **-0.017** | *5.00* | **0.350** | *1.16* | **0.071** | *0.71* | **0.177** | *1.89* |
|  |  |  | 2 |  | 0.4 |  | 2 |  | 2 | **0.878** | *0.27* | **-0.043** | *5.00* | **0.732** | *0.73* | **0.186** | *0.65* | **0.438** | *1.66* |
|  |  |  | 4 |  | 0.8 |  | 4 |  | 4 | **0.890** | *0.51* | **0.019** | *0.83* | **0.881** | *0.92* | **0.610** | *0.50* | **0.600** | *0.69* |
|  |  | 37.5 | 0.25 | 37.5 | 0.05 | 100 | 0.25 | 100 | 0.25 | **0.052** | *4.96* | **0.011** | *0.55* | **0.352** | *1.20* | **0.057** | *0.84* | **0.118** | *1.89* |
|  |  |  | 0.5 |  | 0.1 |  | 0.5 |  | 0.5 | **0.078** | *2.65* | **-0.008** | *5.00* | **0.375** | *1.22* | **0.077** | *0.77* | **0.131** | *2.41* |
|  |  |  | 1 |  | 0.2 |  | 1 |  | 1 | **0.328** | *0.67* | **-0.018** | *5.00* | **0.483** | *1.01* | **0.095** | *0.83* | **0.222** | *1.88* |
|  |  |  | 2 |  | 0.4 |  | 2 |  | 2 | **0.871** | *0.28* | **-0.064** | *5.00* | **0.812** | *0.63* | **0.202** | *0.74* | **0.455** | *1.66* |
|  |  |  | 4 |  | 0.8 |  | 4 |  | 4 | **0.892** | *0.50* | **0.019** | *1.02* | **0.895** | *0.89* | **0.644** | *0.50* | **0.613** | *0.73* |
|  |  | 75 | 0.25 | 75 | 0.05 | 200 | 0.25 | 200 | 0.25 | **0.054** | *5.00* | **0.011** | *1.02* | **0.452** | *1.32* | **0.067** | *1.39* | **0.146** | *2.18* |
|  |  |  | 0.5 |  | 0.1 |  | 0.5 |  | 0.5 | **0.074** | *4.97* | **-0.003** | *5.00* | **0.482** | *1.27* | **0.069** | *1.45* | **0.156** | *3.17* |
|  |  |  | 1 |  | 0.2 |  | 1 |  | 1 | **0.294** | *0.82* | **-0.019** | *5.00* | **0.597** | *0.96* | **0.084** | *1.45* | **0.239** | *2.06* |
|  |  |  | 2 |  | 0.4 |  | 2 |  | 2 | **0.884** | *0.26* | **-0.044** | *5.00* | **0.888** | *0.50* | **0.279** | *0.78* | **0.502** | *1.64* |
|  |  |  | 4 |  | 0.8 |  | 4 |  | 4 | **0.904** | *0.47* | **0.030** | *1.22* | **0.919** | *0.81* | **0.720** | *0.47* | **0.643** | *0.74* |
|  |  | 150 | 0.25 | 150 | 0.05 | 400 | 0.25 | 400 | 0.25 | **0.037** | *5.00* | **0.051** | *1.02* | **0.520** | *1.76* | **0.154** | *1.36* | **0.191** | *2.29* |
|  |  |  | 0.5 |  | 0.1 |  | 0.5 |  | 0.5 | **0.053** | *5.00* | **0.050** | *1.06* | **0.559** | *1.56* | **0.188** | *1.20* | **0.213** | *2.21* |
|  |  |  | 1 |  | 0.2 |  | 1 |  | 1 | **0.314** | *0.90* | **0.027** | *1.46* | **0.664** | *1.11* | **0.337** | *0.79* | **0.335** | *1.06* |
|  |  |  | 2 |  | 0.4 |  | 2 |  | 2 | **0.881** | *0.27* | **0.012** | *2.25* | **0.927** | *0.43* | **0.606** | *0.50* | **0.607** | *0.86* |
|  |  |  | 4 |  | 0.8 |  | 4 |  | 4 | **0.911** | *0.45* | **0.095** | *1.21* | **0.943** | *0.71* | **0.855** | *0.37* | **0.701** | *0.68* |
|  |  | 300 | 0.25 | 300 | 0.05 | 800 | 0.25 | 800 | 0.25 | **0.055** | *5.00* | **0.393** | *0.67* | **0.685** | *1.38* | **0.688** | *0.43* | **0.455** | *1.87* |
|  |  |  | 0.5 |  | 0.1 |  | 0.5 |  | 0.5 | **0.076** | *5.00* | **0.426** | *0.65* | **0.725** | *1.16* | **0.786** | *0.32* | **0.503** | *1.78* |
|  |  |  | 1 |  | 0.2 |  | 1 |  | 1 | **0.498** | *0.53* | **0.379** | *0.75* | **0.822** | *0.74* | **0.906** | *0.19* | **0.651** | *0.55* |
|  |  |  | 2 |  | 0.4 |  | 2 |  | 2 | **0.902** | *0.24* | **0.391** | *0.83* | **0.972** | *0.28* | **0.889** | *0.28* | **0.788** | *0.40* |
|  |  |  | 4 |  | 0.8 |  | 4 |  | 4 | **0.933** | *0.38* | **0.604** | *0.75* | **0.976** | *0.50* | **0.965** | *0.21* | **0.869** | *0.46* |
| **Etoposide** | **SN-38** | 18.8 | 0.0625 | 18.8 | 0.025 | 50 | 0.047 | 50 | 0.047 | **-0.002** | *5.00* | **0.017** | *0.25* | **0.210** | *1.61* | **0.038** | *0.59* | **0.066** | *1.86* |
|  |  |  | 0.125 |  | 0.05 |  | 0.094 |  | 0.094 | **0.021** | *5.00* | **0.006** | *0.43* | **0.237** | *1.53* | **0.049** | *0.59* | **0.078** | *1.89* |
|  |  |  | 0.25 |  | 0.1 |  | 0.188 |  | 0.188 | **0.011** | *5.00* | **0.001** | *1.12* | **0.268** | *1.65* | **0.028** | *1.06* | **0.077** | *2.21* |
|  |  |  | 0.5 |  | 0.2 |  | 0.375 |  | 0.375 | **0.023** | *5.00* | **-0.001** | *5.00* | **0.381** | *1.54* | **0.280** | *0.50* | **0.171** | *3.01* |
|  |  |  | 1 |  | 0.4 |  | 0.75 |  | 0.75 | **0.132** | *0.72* | **0.169** | *0.49* | **0.766** | *0.82* | **0.719** | *0.49* | **0.446** | *0.63* |
|  |  | 37.5 | 0.0625 | 37.5 | 0.025 | 100 | 0.047 | 100 | 0.047 | **0.023** | *5.00* | **0.017** | *0.45* | **0.321** | *1.42* | **0.067** | *0.72* | **0.107** | *1.90* |
|  |  |  | 0.125 |  | 0.05 |  | 0.094 |  | 0.094 | **0.046** | *5.00* | **-0.003** | *5.00* | **0.326** | *1.54* | **0.080** | *0.71* | **0.112** | *3.06* |
|  |  |  | 0.25 |  | 0.1 |  | 0.188 |  | 0.188 | **0.031** | *5.00* | **0.003** | *1.08* | **0.350** | *1.66* | **0.085** | *0.84* | **0.117** | *2.14* |
|  |  |  | 0.5 |  | 0.2 |  | 0.375 |  | 0.375 | **0.015** | *5.00* | **0.029** | *0.63* | **0.434** | *1.61* | **0.371** | *0.49* | **0.212** | *1.93* |
|  |  |  | 1 |  | 0.4 |  | 0.75 |  | 0.75 | **0.165** | *0.75* | **0.187** | *0.54* | **0.746** | *0.93* | **0.740** | *0.50* | **0.459** | *0.68* |
|  |  | 75 | 0.0625 | 75 | 0.025 | 200 | 0.047 | 200 | 0.047 | **0.061** | *5.00* | **0.022** | *0.76* | **0.418** | *1.56* | **0.081** | *1.18* | **0.145** | *2.13* |
|  |  |  | 0.125 |  | 0.05 |  | 0.094 |  | 0.094 | **0.039** | *5.00* | **0.010** | *1.13* | **0.429** | *1.59* | **0.075** | *1.33* | **0.138** | *2.26* |
|  |  |  | 0.25 |  | 0.1 |  | 0.188 |  | 0.188 | **0.078** | *3.93* | **0.023** | *0.87* | **0.435** | *1.79* | **0.137** | *0.98* | **0.168** | *1.89* |
|  |  |  | 0.5 |  | 0.2 |  | 0.375 |  | 0.375 | **0.035** | *5.00* | **0.044** | *0.81* | **0.513** | *1.64* | **0.515** | *0.48* | **0.277** | *1.98* |
|  |  |  | 1 |  | 0.4 |  | 0.75 |  | 0.75 | **0.189** | *0.88* | **0.305** | *0.55* | **0.728** | *1.11* | **0.780** | *0.50* | **0.501** | *0.76* |
|  |  | 150 | 0.0625 | 150 | 0.025 | 400 | 0.047 | 400 | 0.047 | **0.041** | *5.00* | **0.093** | *0.77* | **0.504** | *1.90* | **0.195** | *1.10* | **0.208** | *2.19* |
|  |  |  | 0.125 |  | 0.05 |  | 0.094 |  | 0.094 | **0.036** | *5.00* | **0.082** | *0.85* | **0.514** | *1.90* | **0.309** | *0.76* | **0.235** | *2.13* |
|  |  |  | 0.25 |  | 0.1 |  | 0.188 |  | 0.188 | **0.054** | *5.00* | **0.129** | *0.74* | **0.551** | *1.75* | **0.500** | *0.52* | **0.309** | *2.00* |
|  |  |  | 0.5 |  | 0.2 |  | 0.375 |  | 0.375 | **0.092** | *5.00* | **0.227** | *0.66* | **0.666** | *1.25* | **0.673** | *0.46* | **0.415** | *1.84* |
|  |  |  | 1 |  | 0.4 |  | 0.75 |  | 0.75 | **0.220** | *1.06* | **0.597** | *0.51* | **0.761** | *1.17* | **0.836** | *0.49* | **0.603** | *0.81* |
|  |  | 300 | 0.0625 | 300 | 0.025 | 800 | 0.047 | 800 | 0.047 | **0.091** | *5.00* | **0.529** | *0.53* | **0.685** | *1.35* | **0.674** | *0.46* | **0.495** | *1.83* |
|  |  |  | 0.125 |  | 0.05 |  | 0.094 |  | 0.094 | **0.105** | *5.00* | **0.544** | *0.53* | **0.705** | *1.25* | **0.699** | *0.45* | **0.513** | *1.81* |
|  |  |  | 0.25 |  | 0.1 |  | 0.188 |  | 0.188 | **0.112** | *5.00* | **0.629** | *0.49* | **0.728** | *1.19* | **0.713** | *0.49* | **0.546** | *1.79* |
|  |  |  | 0.5 |  | 0.2 |  | 0.375 |  | 0.375 | **0.138** | *4.52* | **0.760** | *0.44* | **0.760** | *1.17* | **0.776** | *0.51* | **0.608** | *1.66* |
|  |  |  | 1 |  | 0.4 |  | 0.75 |  | 0.75 | **0.312** | *0.86* | **0.929** | *0.35* | **0.769** | *1.48* | **0.860** | *0.55* | **0.718** | *0.81* |
| **Etoposide** | **Vincristine** | 18.8 | 0.625 | 18.8 | 0.0375 | 50 | 0.125 | 50 | 0.188 | **0.958** | *0.41* | **-0.006** | *5.00* | **0.226** | *1.72* | **0.069** | *0.86* | **0.312** | *2.00* |
|  |  |  | 1.25 |  | 0.075 |  | 0.25 |  | 0.375 | **0.968** | *0.72* | **0.001** | *0.91* | **0.222** | *2.30* | **0.111** | *1.07* | **0.326** | *1.25* |
|  |  |  | 2.5 |  | 0.15 |  | 0.5 |  | 0.75 | **0.974** | *1.33* | **0.000** | *5.00* | **0.273** | *2.32* | **0.514** | *0.68* | **0.440** | *2.33* |
|  |  |  | 5 |  | 0.3 |  | 1 |  | 1.5 | **0.968** | *2.91* | **0.022** | *0.85* | **0.430** | *1.49* | **0.923** | *0.42* | **0.586** | *1.42* |
|  |  |  | 10 |  | 0.6 |  | 2 |  | 3 | **0.967** | *5.00* | **0.515** | *0.68* | **0.583** | *1.16* | **0.985** | *0.39* | **0.762** | *1.81* |
|  |  | 37.5 | 0.625 | 37.5 | 0.0375 | 100 | 0.125 | 100 | 0.188 | **0.959** | *0.41* | **-0.013** | *5.00* | **0.332** | *1.43* | **0.084** | *1.03* | **0.341** | *1.97* |
|  |  |  | 1.25 |  | 0.075 |  | 0.25 |  | 0.375 | **0.972** | *0.69* | **-0.005** | *5.00* | **0.333** | *1.67* | **0.115** | *1.26* | **0.354** | *2.16* |
|  |  |  | 2.5 |  | 0.15 |  | 0.5 |  | 0.75 | **0.974** | *1.33* | **-0.024** | *5.00* | **0.365** | *1.80* | **0.498** | *0.75* | **0.453** | *2.22* |
|  |  |  | 5 |  | 0.3 |  | 1 |  | 1.5 | **0.968** | *2.92* | **0.006** | *1.51* | **0.447** | *1.67* | **0.913** | *0.46* | **0.583** | *1.64* |
|  |  |  | 10 |  | 0.6 |  | 2 |  | 3 | **0.968** | *5.00* | **0.504** | *0.72* | **0.594** | *1.23* | **0.981** | *0.42* | **0.762** | *1.84* |
|  |  | 75 | 0.625 | 75 | 0.0375 | 200 | 0.125 | 200 | 0.188 | **0.953** | *0.43* | **-0.036** | *5.00* | **0.423** | *1.55* | **0.086** | *1.55* | **0.357** | *2.13* |
|  |  |  | 1.25 |  | 0.075 |  | 0.25 |  | 0.375 | **0.973** | *0.68* | **-0.022** | *5.00* | **0.436** | *1.58* | **0.114** | *1.67* | **0.375** | *2.23* |
|  |  |  | 2.5 |  | 0.15 |  | 0.5 |  | 0.75 | **0.975** | *1.32* | **-0.032** | *5.00* | **0.431** | *1.91* | **0.518** | *0.81* | **0.473** | *2.26* |
|  |  |  | 5 |  | 0.3 |  | 1 |  | 1.5 | **0.968** | *2.90* | **0.016** | *1.55* | **0.497** | *1.74* | **0.885** | *0.55* | **0.592** | *1.68* |
|  |  |  | 10 |  | 0.6 |  | 2 |  | 3 | **0.968** | *5.00* | **0.381** | *0.89* | **0.616** | *1.33* | **0.976** | *0.48* | **0.735** | *1.92* |
|  |  | 150 | 0.625 | 150 | 0.0375 | 400 | 0.125 | 400 | 0.188 | **0.953** | *0.43* | **0.028** | *1.37* | **0.513** | *1.81* | **0.106** | *2.21* | **0.400** | *1.46* |
|  |  |  | 1.25 |  | 0.075 |  | 0.25 |  | 0.375 | **0.972** | *0.68* | **0.026** | *1.51* | **0.508** | *1.95* | **0.204** | *1.62* | **0.427** | *1.44* |
|  |  |  | 2.5 |  | 0.15 |  | 0.5 |  | 0.75 | **0.973** | *1.35* | **0.020** | *1.85* | **0.513** | *2.08* | **0.559** | *0.89* | **0.516** | *1.54* |
|  |  |  | 5 |  | 0.3 |  | 1 |  | 1.5 | **0.966** | *2.98* | **0.029** | *1.91* | **0.499** | *2.65* | **0.859** | *0.66* | **0.588** | *2.05* |
|  |  |  | 10 |  | 0.6 |  | 2 |  | 3 | **0.965** | *5.00* | **0.332** | *1.11* | **0.529** | *2.94* | **0.960** | *0.63* | **0.696** | *2.42* |
|  |  | 300 | 0.625 | 300 | 0.0375 | 800 | 0.125 | 800 | 0.188 | **0.957** | *0.42* | **0.373** | *0.72* | **0.672** | *1.44* | **0.566** | *0.75* | **0.642** | *0.83* |
|  |  |  | 1.25 |  | 0.075 |  | 0.25 |  | 0.375 | **0.974** | *0.67* | **0.361** | *0.78* | **0.680** | *1.41* | **0.596** | *0.82* | **0.653** | *0.92* |
|  |  |  | 2.5 |  | 0.15 |  | 0.5 |  | 0.75 | **0.972** | *1.37* | **0.363** | *0.87* | **0.670** | *1.57* | **0.704** | *0.82* | **0.677** | *1.16* |
|  |  |  | 5 |  | 0.3 |  | 1 |  | 1.5 | **0.968** | *2.90* | **0.357** | *1.06* | **0.663** | *1.80* | **0.789** | *0.99* | **0.694** | *1.69* |
|  |  |  | 10 |  | 0.6 |  | 2 |  | 3 | **0.964** | *5.00* | **0.665** | *0.97* | **0.663** | *2.12* | **0.896** | *1.11* | **0.797** | *2.30* |
| **Romidepsin** | **Doxorubicin** | 0.25 | 10 | 0.05 | 37.5 | 0.25 | 5 | 0.25 | 10 | **0.097** | *0.44* | **0.240** | *0.52* | **0.157** | *0.77* | **0.029** | *0.33* | **0.131** | *0.52* |
|  |  |  | 20 |  | 75 |  | 10 |  | 20 | **0.115** | *0.48* | **0.880** | *0.31* | **0.313** | *0.58* | **0.064** | *0.34* | **0.343** | *0.43* |
|  |  |  | 40 |  | 150 |  | 20 |  | 40 | **0.339** | *0.31* | **0.972** | *0.34* | **0.461** | *0.54* | **0.054** | *0.64* | **0.456** | *0.46* |
|  |  |  | 80 |  | 300 |  | 40 |  | 80 | **0.451** | *0.37* | **0.989** | *0.45* | **0.592** | *0.57* | **0.102** | *0.80* | **0.534** | *0.55* |
|  |  |  | 160 |  | 600 |  | 80 |  | 160 | **0.402** | *0.69* | **0.995** | *0.64* | **0.726** | *0.57* | **0.588** | *0.38* | **0.678** | *0.57* |
|  |  | 0.5 | 10 | 0.1 | 37.5 | 0.5 | 5 | 0.5 | 10 | **0.052** | *1.15* | **0.334** | *0.46* | **0.191** | *0.85* | **0.045** | *0.39* | **0.155** | *0.71* |
|  |  |  | 20 |  | 75 |  | 10 |  | 20 | **0.138** | *0.71* | **0.900** | *0.30* | **0.383** | *0.59* | **0.065** | *0.44* | **0.372** | *0.51* |
|  |  |  | 40 |  | 150 |  | 20 |  | 40 | **0.475** | *0.34* | **0.976** | *0.33* | **0.497** | *0.60* | **0.062** | *0.70* | **0.502** | *0.49* |
|  |  |  | 80 |  | 300 |  | 40 |  | 80 | **0.578** | *0.37* | **0.989** | *0.46* | **0.634** | *0.58* | **0.153** | *0.69* | **0.589** | *0.52* |
|  |  |  | 160 |  | 600 |  | 80 |  | 160 | **0.616** | *0.52* | **0.996** | *0.61* | **0.769** | *0.53* | **0.726** | *0.29* | **0.777** | *0.49* |
|  |  | 1 | 10 | 0.2 | 37.5 | 1 | 5 | 1 | 10 | **0.295** | *0.70* | **0.356** | *0.49* | **0.318** | *0.86* | **0.111** | *0.41* | **0.270** | *0.62* |
|  |  |  | 20 |  | 75 |  | 10 |  | 20 | **0.484** | *0.48* | **0.910** | *0.31* | **0.532** | *0.62* | **0.115** | *0.49* | **0.510** | *0.48* |
|  |  |  | 40 |  | 150 |  | 20 |  | 40 | **0.804** | *0.24* | **0.975** | *0.35* | **0.651** | *0.56* | **0.110** | *0.67* | **0.635** | *0.46* |
|  |  |  | 80 |  | 300 |  | 40 |  | 80 | **0.918** | *0.17* | **0.991** | *0.46* | **0.757** | *0.52* | **0.300** | *0.54* | **0.742** | *0.42* |
|  |  |  | 160 |  | 600 |  | 80 |  | 160 | **0.945** | *0.19* | **0.996** | *0.62* | **0.857** | *0.45* | **0.898** | *0.18* | **0.924** | *0.36* |
|  |  | 2 | 10 | 0.4 | 37.5 | 2 | 5 | 2 | 10 | **0.882** | *0.28* | **0.374** | *0.57* | **0.789** | *0.61* | **0.238** | *0.50* | **0.571** | *0.49* |
|  |  |  | 20 |  | 75 |  | 10 |  | 20 | **0.901** | *0.26* | **0.920** | *0.36* | **0.896** | *0.45* | **0.264** | *0.52* | **0.745** | *0.39* |
|  |  |  | 40 |  | 150 |  | 20 |  | 40 | **0.929** | *0.23* | **0.985** | *0.33* | **0.945** | *0.34* | **0.329** | *0.53* | **0.797** | *0.36* |
|  |  |  | 80 |  | 300 |  | 40 |  | 80 | **0.968** | *0.16* | **0.995** | *0.39* | **0.966** | *0.29* | **0.654** | *0.36* | **0.896** | *0.30* |
|  |  |  | 160 |  | 600 |  | 80 |  | 160 | **0.985** | *0.13* | **0.998** | *0.54* | **0.977** | *0.26* | **0.925** | *0.20* | **0.971** | *0.28* |
|  |  | 4 | 10 | 0.8 | 37.5 | 4 | 5 | 4 | 10 | **0.912** | *0.45* | **0.550** | *0.61* | **0.934** | *0.71* | **0.687** | *0.42* | **0.771** | *0.55* |
|  |  |  | 20 |  | 75 |  | 10 |  | 20 | **0.932** | *0.39* | **0.960** | *0.38* | **0.953** | *0.62* | **0.710** | *0.42* | **0.889** | *0.45* |
|  |  |  | 40 |  | 150 |  | 20 |  | 40 | **0.956** | *0.32* | **0.993** | *0.33* | **0.976** | *0.47* | **0.783** | *0.38* | **0.927** | *0.38* |
|  |  |  | 80 |  | 300 |  | 40 |  | 80 | **0.982** | *0.21* | **0.998** | *0.36* | **0.991** | *0.32* | **0.891** | *0.30* | **0.965** | *0.30* |
|  |  |  | 160 |  | 600 |  | 80 |  | 160 | **0.997** | *0.09* | **0.998** | *0.53* | **0.996** | *0.24* | **0.966** | *0.21* | **0.989** | *0.27* |
| **Romidepsin** | **SN-38** | 0.25 | 0.0625 | 0.05 | 0.025 | 0.25 | 0.047 | 0.25 | 0.047 | **0.058** | *0.51* | **-0.020** | *5.00* | **0.044** | *1.10* | **0.035** | *0.24* | **0.029** | *1.71* |
|  |  |  | 0.125 |  | 0.05 |  | 0.094 |  | 0.094 | **0.075** | *0.47* | **-0.047** | *5.00* | **0.099** | *1.10* | **0.006** | *0.63* | **0.033** | *1.80* |
|  |  |  | 0.25 |  | 0.1 |  | 0.188 |  | 0.188 | **0.042** | *0.73* | **-0.042** | *5.00* | **0.184** | *1.25* | **0.032** | *0.55* | **0.054** | *1.88* |
|  |  |  | 0.5 |  | 0.2 |  | 0.375 |  | 0.375 | **-0.007** | *5.00* | **-0.044** | *5.00* | **0.363** | *1.31* | **0.296** | *0.45* | **0.152** | *2.94* |
|  |  |  | 1 |  | 0.4 |  | 0.75 |  | 0.75 | **0.120** | *0.74* | **0.143** | *0.46* | **0.821** | *0.69* | **0.806** | *0.43* | **0.473** | *0.58* |
|  |  | 0.5 | 0.0625 | 0.1 | 0.025 | 0.5 | 0.047 | 0.5 | 0.047 | **0.071** | *0.88* | **-0.027** | *5.00* | **0.060** | *1.29* | **0.033** | *0.38* | **0.034** | *1.89* |
|  |  |  | 0.125 |  | 0.05 |  | 0.094 |  | 0.094 | **0.074** | *0.89* | **-0.045** | *5.00* | **0.110** | *1.31* | **0.040** | *0.45* | **0.045** | *1.91* |
|  |  |  | 0.25 |  | 0.1 |  | 0.188 |  | 0.188 | **0.050** | *1.18* | **-0.064** | *5.00* | **0.219** | *1.31* | **0.044** | *0.62* | **0.062** | *2.03* |
|  |  |  | 0.5 |  | 0.2 |  | 0.375 |  | 0.375 | **0.024** | *1.92* | **-0.049** | *5.00* | **0.380** | *1.40* | **0.343** | *0.46* | **0.175** | *2.20* |
|  |  |  | 1 |  | 0.4 |  | 0.75 |  | 0.75 | **0.181** | *0.85* | **0.134** | *0.50* | **0.830** | *0.73* | **0.891** | *0.36* | **0.509** | *0.61* |
|  |  | 1 | 0.0625 | 0.2 | 0.025 | 1 | 0.047 | 1 | 0.047 | **0.397** | *0.53* | **-0.062** | *5.00* | **0.104** | *1.53* | **0.056** | *0.53* | **0.123** | *1.90* |
|  |  |  | 0.125 |  | 0.05 |  | 0.094 |  | 0.094 | **0.373** | *0.58* | **-0.069** | *5.00* | **0.151** | *1.56* | **0.044** | *0.68* | **0.125** | *1.95* |
|  |  |  | 0.25 |  | 0.1 |  | 0.188 |  | 0.188 | **0.405** | *0.57* | **-0.086** | *5.00* | **0.312** | *1.34* | **0.065** | *0.75* | **0.174** | *1.92* |
|  |  |  | 0.5 |  | 0.2 |  | 0.375 |  | 0.375 | **0.441** | *0.60* | **-0.081** | *5.00* | **0.515** | *1.26* | **0.493** | *0.45* | **0.342** | *1.83* |
|  |  |  | 1 |  | 0.4 |  | 0.75 |  | 0.75 | **0.718** | *0.44* | **0.119** | *0.57* | **0.840** | *0.83* | **0.962** | *0.28* | **0.660** | *0.53* |
|  |  | 2 | 0.0625 | 0.4 | 0.025 | 2 | 0.047 | 2 | 0.047 | **0.886** | *0.27* | **-0.117** | *5.00* | **0.578** | *0.96* | **0.246** | *0.50* | **0.398** | *1.68* |
|  |  |  | 0.125 |  | 0.05 |  | 0.094 |  | 0.094 | **0.888** | *0.28* | **-0.118** | *5.00* | **0.607** | *0.99* | **0.258** | *0.54* | **0.409** | *1.70* |
|  |  |  | 0.25 |  | 0.1 |  | 0.188 |  | 0.188 | **0.879** | *0.31* | **-0.093** | *5.00* | **0.647** | *1.06* | **0.302** | *0.59* | **0.434** | *1.74* |
|  |  |  | 0.5 |  | 0.2 |  | 0.375 |  | 0.375 | **0.882** | *0.34* | **-0.085** | *5.00* | **0.728** | *1.11* | **0.652** | *0.47* | **0.544** | *1.73* |
|  |  |  | 1 |  | 0.4 |  | 0.75 |  | 0.75 | **0.891** | *0.39* | **0.128** | *0.67* | **0.819** | *1.18* | **0.933** | *0.38* | **0.693** | *0.66* |
|  |  | 4 | 0.0625 | 0.8 | 0.025 | 4 | 0.047 | 4 | 0.047 | **0.896** | *0.50* | **-0.009** | *5.00* | **0.876** | *0.95* | **0.677** | *0.45* | **0.610** | *1.73* |
|  |  |  | 0.125 |  | 0.05 |  | 0.094 |  | 0.094 | **0.899** | *0.50* | **-0.019** | *5.00* | **0.874** | *0.99* | **0.696** | *0.46* | **0.613** | *1.74* |
|  |  |  | 0.25 |  | 0.1 |  | 0.188 |  | 0.188 | **0.905** | *0.50* | **-0.030** | *5.00* | **0.870** | *1.07* | **0.753** | *0.47* | **0.624** | *1.76* |
|  |  |  | 0.5 |  | 0.2 |  | 0.375 |  | 0.375 | **0.909** | *0.52* | **0.023** | *0.94* | **0.864** | *1.22* | **0.903** | *0.39* | **0.675** | *0.77* |
|  |  |  | 1 |  | 0.4 |  | 0.75 |  | 0.75 | **0.927** | *0.52* | **0.363** | *0.70* | **0.877** | *1.40* | **0.977** | *0.33* | **0.786** | *0.74* |
| **Romidepsin** | **Vincristine** | 0.25 | 0.125 | 0.05 | 0.0375 | 0.25 | 0.125 | 0.25 | 0.188 | **0.920** | *0.06* | **0.016** | *0.13* | **0.049** | *5.00* | **0.064** | *0.68* | **0.262** | *1.47* |
|  |  |  | 0.25 |  | 0.075 |  | 0.25 |  | 0.375 | **0.960** | *0.08* | **0.024** | *0.20* | **0.065** | *5.00* | **0.125** | *0.89* | **0.293** | *1.54* |
|  |  |  | 0.5 |  | 0.15 |  | 0.5 |  | 0.75 | **0.963** | *0.14* | **-0.004** | *5.00* | **0.215** | *2.42* | **0.626** | *0.55* | **0.450** | *2.03* |
|  |  |  | 1 |  | 0.3 |  | 1 |  | 1.5 | **0.971** | *0.25* | **0.042** | *0.62* | **0.469** | *1.06* | **0.965** | *0.29* | **0.612** | *0.56* |
|  |  |  | 2 |  | 0.6 |  | 2 |  | 3 | **0.967** | *0.51* | **0.647** | *0.61* | **0.639** | *0.84* | **0.989** | *0.33* | **0.811** | *0.57* |
|  |  | 0.5 | 0.125 | 0.1 | 0.0375 | 0.5 | 0.125 | 0.5 | 0.188 | **0.953** | *0.06* | **0.003** | *0.23* | **0.060** | *4.52* | **0.073** | *0.73* | **0.272** | *1.39* |
|  |  |  | 0.25 |  | 0.075 |  | 0.25 |  | 0.375 | **0.973** | *0.08* | **-0.005** | *5.00* | **0.071** | *5.00* | **0.132** | *0.94* | **0.293** | *2.75* |
|  |  |  | 0.5 |  | 0.15 |  | 0.5 |  | 0.75 | **0.981** | *0.12* | **0.004** | *0.57* | **0.215** | *2.62* | **0.610** | *0.59* | **0.453** | *0.97* |
|  |  |  | 1 |  | 0.3 |  | 1 |  | 1.5 | **0.978** | *0.24* | **0.042** | *0.66* | **0.499** | *1.04* | **0.968** | *0.29* | **0.622** | *0.56* |
|  |  |  | 2 |  | 0.6 |  | 2 |  | 3 | **0.980** | *0.44* | **0.636** | *0.63* | **0.671** | *0.80* | **0.992** | *0.30* | **0.820** | *0.54* |
|  |  | 1 | 0.125 | 0.2 | 0.0375 | 1 | 0.125 | 1 | 0.188 | **0.961** | *0.08* | **-0.026** | *5.00* | **0.106** | *2.81* | **0.109** | *0.77* | **0.288** | *2.16* |
|  |  |  | 0.25 |  | 0.075 |  | 0.25 |  | 0.375 | **0.980** | *0.08* | **-0.036** | *5.00* | **0.113** | *4.15* | **0.180** | *0.92* | **0.309** | *2.54* |
|  |  |  | 0.5 |  | 0.15 |  | 0.5 |  | 0.75 | **0.983** | *0.13* | **-0.027** | *5.00* | **0.236** | *2.66* | **0.610** | *0.65* | **0.451** | *2.11* |
|  |  |  | 1 |  | 0.3 |  | 1 |  | 1.5 | **0.977** | *0.25* | **0.003** | *1.19* | **0.633** | *0.78* | **0.975** | *0.27* | **0.647** | *0.62* |
|  |  |  | 2 |  | 0.6 |  | 2 |  | 3 | **0.980** | *0.45* | **0.565** | *0.71* | **0.788** | *0.58* | **0.991** | *0.32* | **0.831** | *0.51* |
|  |  | 2 | 0.125 | 0.4 | 0.0375 | 2 | 0.125 | 2 | 0.188 | **0.951** | *0.14* | **-0.073** | *5.00* | **0.573** | *0.95* | **0.254** | *0.71* | **0.426** | *1.70* |
|  |  |  | 0.25 |  | 0.075 |  | 0.25 |  | 0.375 | **0.960** | *0.16* | **-0.063** | *5.00* | **0.587** | *0.99* | **0.414** | *0.71* | **0.475** | *1.71* |
|  |  |  | 0.5 |  | 0.15 |  | 0.5 |  | 0.75 | **0.959** | *0.23* | **-0.069** | *5.00* | **0.662** | *0.92* | **0.737** | *0.59* | **0.572** | *1.69* |
|  |  |  | 1 |  | 0.3 |  | 1 |  | 1.5 | **0.969** | *0.32* | **-0.022** | *5.00* | **0.880** | *0.51* | **0.962** | *0.37* | **0.697** | *1.55* |
|  |  |  | 2 |  | 0.6 |  | 2 |  | 3 | **0.968** | *0.57* | **0.486** | *0.83* | **0.931** | *0.41* | **0.984** | *0.44* | **0.842** | *0.56* |
|  |  | 4 | 0.125 | 0.8 | 0.0375 | 4 | 0.125 | 4 | 0.188 | **0.960** | *0.22* | **0.007** | *0.86* | **0.906** | *0.83* | **0.752** | *0.45* | **0.656** | *0.59* |
|  |  |  | 0.25 |  | 0.075 |  | 0.25 |  | 0.375 | **0.966** | *0.23* | **-0.007** | *5.00* | **0.907** | *0.83* | **0.861** | *0.41* | **0.682** | *1.62* |
|  |  |  | 0.5 |  | 0.15 |  | 0.5 |  | 0.75 | **0.965** | *0.30* | **-0.007** | *5.00* | **0.918** | *0.79* | **0.961** | *0.30* | **0.709** | *1.60* |
|  |  |  | 1 |  | 0.3 |  | 1 |  | 1.5 | **0.969** | *0.40* | **0.035** | *1.19* | **0.935** | *0.73* | **0.987** | *0.27* | **0.732** | *0.65* |
|  |  |  | 2 |  | 0.6 |  | 2 |  | 3 | **0.976** | *0.58* | **0.590** | *0.92* | **0.948** | *0.68* | **0.992** | *0.35* | **0.877** | *0.63* |
| **SN-38** | **Doxorubicin** | 0.0625 | 10 | 0.025 | 37.5 | 0.047 | 5 | 0.047 | 10 | **0.050** | *0.17* | **0.254** | *0.51* | **0.185** | *0.69* | **0.021** | *0.35* | **0.127** | *0.43* |
|  |  |  | 20 |  | 75 |  | 10 |  | 20 | **0.104** | *0.21* | **0.891** | *0.30* | **0.303** | *0.60* | **0.043** | *0.40* | **0.335** | *0.37* |
|  |  |  | 40 |  | 150 |  | 20 |  | 40 | **0.376** | *0.17* | **0.977** | *0.31* | **0.428** | *0.60* | **0.046** | *0.67* | **0.457** | *0.43* |
|  |  |  | 80 |  | 300 |  | 40 |  | 80 | **0.421** | *0.29* | **0.990** | *0.44* | **0.541** | *0.67* | **0.076** | *0.93* | **0.507** | *0.58* |
|  |  |  | 160 |  | 600 |  | 80 |  | 160 | **0.396** | *0.59* | **0.996** | *0.60* | **0.686** | *0.66* | **0.398** | *0.57* | **0.619** | *0.61* |
|  |  | 0.125 | 10 | 0.05 | 37.5 | 0.094 | 5 | 0.094 | 10 | **0.048** | *0.21* | **0.357** | *0.44* | **0.246** | *0.70* | **0.051** | *0.32* | **0.175** | *0.42* |
|  |  |  | 20 |  | 75 |  | 10 |  | 20 | **0.135** | *0.20* | **0.923** | *0.27* | **0.355** | *0.63* | **0.061** | *0.41* | **0.368** | *0.38* |
|  |  |  | 40 |  | 150 |  | 20 |  | 40 | **0.436** | *0.17* | **0.981** | *0.30* | **0.462** | *0.63* | **0.061** | *0.66* | **0.485** | *0.44* |
|  |  |  | 80 |  | 300 |  | 40 |  | 80 | **0.431** | *0.30* | **0.992** | *0.42* | **0.557** | *0.72* | **0.102** | *0.86* | **0.520** | *0.57* |
|  |  |  | 160 |  | 600 |  | 80 |  | 160 | **0.405** | *0.60* | **0.996** | *0.62* | **0.742** | *0.55* | **0.448** | *0.56* | **0.648** | *0.58* |
|  |  | 0.25 | 10 | 0.1 | 37.5 | 0.188 | 5 | 0.188 | 10 | **0.047** | *0.28* | **0.410** | *0.45* | **0.338** | *0.80* | **0.078** | *0.42* | **0.218** | *0.49* |
|  |  |  | 20 |  | 75 |  | 10 |  | 20 | **0.155** | *0.24* | **0.904** | *0.32* | **0.396** | *0.80* | **0.083** | *0.51* | **0.384** | *0.47* |
|  |  |  | 40 |  | 150 |  | 20 |  | 40 | **0.461** | *0.19* | **0.983** | *0.30* | **0.483** | *0.80* | **0.073** | *0.76* | **0.500** | *0.51* |
|  |  |  | 80 |  | 300 |  | 40 |  | 80 | **0.433** | *0.33* | **0.990** | *0.46* | **0.587** | *0.80* | **0.173** | *0.75* | **0.546** | *0.58* |
|  |  |  | 160 |  | 600 |  | 80 |  | 160 | **0.416** | *0.62* | **0.996** | *0.63* | **0.715** | *0.74* | **0.501** | *0.57* | **0.657** | *0.64* |
|  |  | 0.5 | 10 | 0.2 | 37.5 | 0.375 | 5 | 0.375 | 10 | **0.050** | *0.41* | **0.513** | *0.46* | **0.458** | *1.00* | **0.207** | *0.51* | **0.307** | *0.60* |
|  |  |  | 20 |  | 75 |  | 10 |  | 20 | **0.192** | *0.31* | **0.956** | *0.28* | **0.482** | *1.03* | **0.253** | *0.52* | **0.471** | *0.53* |
|  |  |  | 40 |  | 150 |  | 20 |  | 40 | **0.489** | *0.25* | **0.984** | *0.33* | **0.550** | *0.99* | **0.300** | *0.56* | **0.581** | *0.53* |
|  |  |  | 80 |  | 300 |  | 40 |  | 80 | **0.427** | *0.40* | **0.992** | *0.45* | **0.645** | *0.92* | **0.441** | *0.57* | **0.626** | *0.59* |
|  |  |  | 160 |  | 600 |  | 80 |  | 160 | **0.437** | *0.66* | **0.996** | *0.65* | **0.744** | *0.85* | **0.580** | *0.63* | **0.689** | *0.70* |
|  |  | 1 | 10 | 0.4 | 37.5 | 0.75 | 5 | 0.75 | 10 | **0.200** | *0.42* | **0.787** | *0.43* | **0.814** | *0.66* | **0.695** | *0.50* | **0.624** | *0.50* |
|  |  |  | 20 |  | 75 |  | 10 |  | 20 | **0.331** | *0.39* | **0.968** | *0.33* | **0.789** | *0.75* | **0.687** | *0.52* | **0.694** | *0.50* |
|  |  |  | 40 |  | 150 |  | 20 |  | 40 | **0.491** | *0.37* | **0.984** | *0.39* | **0.758** | *0.89* | **0.709** | *0.54* | **0.736** | *0.55* |
|  |  |  | 80 |  | 300 |  | 40 |  | 80 | **0.448** | *0.52* | **0.992** | *0.50* | **0.739** | *1.07* | **0.720** | *0.59* | **0.725** | *0.67* |
|  |  |  | 160 |  | 600 |  | 80 |  | 160 | **0.453** | *0.77* | **0.996** | *0.69* | **0.762** | *1.17* | **0.750** | *0.67* | **0.740** | *0.83* |
| **SN-38** | **Vincristine** | 0.0625 | 0.125 | 0.025 | 0.0375 | 0.047 | 0.125 | 0.047 | 0.188 | **0.928** | *0.05* | **0.010** | *0.14* | **0.072** | *3.46* | **0.078** | *0.59* | **0.272** | *1.06* |
|  |  |  | 0.25 |  | 0.075 |  | 0.25 |  | 0.375 | **0.957** | *0.07* | **0.011** | *0.24* | **0.092** | *4.56* | **0.131** | *0.85* | **0.298** | *1.43* |
|  |  |  | 0.5 |  | 0.15 |  | 0.5 |  | 0.75 | **0.955** | *0.14* | **0.020** | *0.38* | **0.217** | *2.41* | **0.559** | *0.62* | **0.438** | *0.89* |
|  |  |  | 1 |  | 0.3 |  | 1 |  | 1.5 | **0.959** | *0.27* | **0.034** | *0.65* | **0.449** | *1.15* | **0.931** | *0.41* | **0.593** | *0.62* |
|  |  |  | 2 |  | 0.6 |  | 2 |  | 3 | **0.954** | *0.56* | **0.652** | *0.60* | **0.587** | *1.08* | **0.983** | *0.42* | **0.794** | *0.67* |
|  |  | 0.125 | 0.125 | 0.05 | 0.0375 | 0.094 | 0.125 | 0.094 | 0.188 | **0.924** | *0.05* | **0.007** | *0.20* | **0.118** | *2.15* | **0.050** | *0.82* | **0.275** | *0.81* |
|  |  |  | 0.25 |  | 0.075 |  | 0.25 |  | 0.375 | **0.952** | *0.08* | **0.002** | *0.39* | **0.147** | *2.65* | **0.115** | *0.98* | **0.304** | *1.03* |
|  |  |  | 0.5 |  | 0.15 |  | 0.5 |  | 0.75 | **0.960** | *0.14* | **-0.001** | *5.00* | **0.252** | *2.08* | **0.537** | *0.69* | **0.437** | *1.98* |
|  |  |  | 1 |  | 0.3 |  | 1 |  | 1.5 | **0.958** | *0.28* | **0.031** | *0.70* | **0.456** | *1.23* | **0.915** | *0.47* | **0.590** | *0.67* |
|  |  |  | 2 |  | 0.6 |  | 2 |  | 3 | **0.954** | *0.57* | **0.585** | *0.65* | **0.613** | *1.01* | **0.980** | *0.46* | **0.783** | *0.67* |
|  |  | 0.25 | 0.125 | 0.1 | 0.0375 | 0.188 | 0.125 | 0.188 | 0.188 | **0.924** | *0.06* | **-0.007** | *5.00* | **0.225** | *1.39* | **0.059** | *0.93* | **0.300** | *1.85* |
|  |  |  | 0.25 |  | 0.075 |  | 0.25 |  | 0.375 | **0.954** | *0.09* | **-0.006** | *5.00* | **0.237** | *1.78* | **0.124** | *1.08* | **0.327** | *1.99* |
|  |  |  | 0.5 |  | 0.15 |  | 0.5 |  | 0.75 | **0.956** | *0.16* | **-0.015** | *5.00* | **0.321** | *1.72* | **0.500** | *0.81* | **0.441** | *1.92* |
|  |  |  | 1 |  | 0.3 |  | 1 |  | 1.5 | **0.955** | *0.30* | **0.015** | *0.90* | **0.489** | *1.26* | **0.887** | *0.59* | **0.587** | *0.76* |
|  |  |  | 2 |  | 0.6 |  | 2 |  | 3 | **0.951** | *0.59* | **0.481** | *0.75* | **0.640** | *1.02* | **0.967** | *0.61* | **0.760** | *0.74* |
|  |  | 0.5 | 0.125 | 0.2 | 0.0375 | 0.375 | 0.125 | 0.375 | 0.188 | **0.912** | *0.09* | **-0.019** | *5.00* | **0.417** | *1.15* | **0.241** | *0.71* | **0.388** | *1.74* |
|  |  |  | 0.25 |  | 0.075 |  | 0.25 |  | 0.375 | **0.948** | *0.11* | **-0.016** | *5.00* | **0.409** | *1.34* | **0.397** | *0.75* | **0.435** | *1.80* |
|  |  |  | 0.5 |  | 0.15 |  | 0.5 |  | 0.75 | **0.949** | *0.18* | **-0.030** | *5.00* | **0.453** | *1.42* | **0.672** | *0.72* | **0.511** | *1.83* |
|  |  |  | 1 |  | 0.3 |  | 1 |  | 1.5 | **0.947** | *0.33* | **0.003** | *1.43* | **0.554** | *1.30* | **0.860** | *0.75* | **0.591** | *0.95* |
|  |  |  | 2 |  | 0.6 |  | 2 |  | 3 | **0.942** | *0.65* | **0.364** | *0.90* | **0.697** | *1.01* | **0.948** | *0.82* | **0.737** | *0.84* |
|  |  | 1 | 0.125 | 0.4 | 0.0375 | 0.75 | 0.125 | 0.75 | 0.188 | **0.892** | *0.15* | **0.110** | *0.52* | **0.838** | *0.59* | **0.702** | *0.59* | **0.635** | *0.46* |
|  |  |  | 0.25 |  | 0.075 |  | 0.25 |  | 0.375 | **0.920** | *0.17* | **0.107** | *0.59* | **0.831** | *0.62* | **0.744** | *0.65* | **0.651** | *0.51* |
|  |  |  | 0.5 |  | 0.15 |  | 0.5 |  | 0.75 | **0.926** | *0.25* | **0.102** | *0.72* | **0.817** | *0.69* | **0.797** | *0.76* | **0.661** | *0.60* |
|  |  |  | 1 |  | 0.3 |  | 1 |  | 1.5 | **0.921** | *0.43* | **0.118** | *0.94* | **0.794** | *0.83* | **0.847** | *0.97* | **0.670** | *0.79* |
|  |  |  | 2 |  | 0.6 |  | 2 |  | 3 | **0.924** | *0.75* | **0.493** | *0.97* | **0.827** | *0.81* | **0.896** | *1.29* | **0.785** | *0.95* |
| **SP2509** | **Doxorubicin** | 62.5 | 10 | 62.5 | 37.5 | 125 | 5 | 157 | 10 | **-0.090** | *5.00* | **0.336** | *0.51* | **0.142** | *1.39* | **0.016** | *0.36* | **0.101** | *1.81* |
|  |  |  | 20 |  | 75 |  | 10 |  | 20 | **0.042** | *0.91* | **0.899** | *0.30* | **0.290** | *0.81* | **0.025** | *0.50* | **0.314** | *0.63* |
|  |  |  | 40 |  | 150 |  | 20 |  | 40 | **0.352** | *0.24* | **0.973** | *0.33* | **0.429** | *0.68* | **0.042** | *0.68* | **0.449** | *0.48* |
|  |  |  | 80 |  | 300 |  | 40 |  | 80 | **0.348** | *0.40* | **0.989** | *0.46* | **0.561** | *0.66* | **0.106** | *0.76* | **0.501** | *0.57* |
|  |  |  | 160 |  | 600 |  | 80 |  | 160 | **0.403** | *0.64* | **0.997** | *0.51* | **0.704** | *0.61* | **0.594** | *0.38* | **0.675** | *0.54* |
|  |  | 125 | 10 | 125 | 37.5 | 250 | 5 | 313 | 10 | **0.184** | *0.40* | **0.438** | *0.50* | **0.219** | *1.32* | **0.016** | *0.45* | **0.214** | *0.67* |
|  |  |  | 20 |  | 75 |  | 10 |  | 20 | **0.208** | *0.41* | **0.890** | *0.33* | **0.354** | *0.87* | **0.022** | *0.61* | **0.368** | *0.56* |
|  |  |  | 40 |  | 150 |  | 20 |  | 40 | **0.395** | *0.29* | **0.954** | *0.42* | **0.481** | *0.71* | **0.049** | *0.70* | **0.470** | *0.53* |
|  |  |  | 80 |  | 300 |  | 40 |  | 80 | **0.388** | *0.44* | **0.987** | *0.50* | **0.618** | *0.62* | **0.201** | *0.57* | **0.548** | *0.53* |
|  |  |  | 160 |  | 600 |  | 80 |  | 160 | **0.476** | *0.60* | **0.997** | *0.54* | **0.721** | *0.63* | **0.616** | *0.40* | **0.703** | *0.54* |
|  |  | 250 | 10 | 250 | 37.5 | 500 | 5 | 625 | 10 | **0.434** | *0.29* | **0.635** | *0.46* | **0.363** | *1.20* | **0.019** | *0.58* | **0.363** | *0.63* |
|  |  |  | 20 |  | 75 |  | 10 |  | 20 | **0.442** | *0.31* | **0.849** | *0.44* | **0.444** | *0.99* | **0.025** | *0.73* | **0.440** | *0.62* |
|  |  |  | 40 |  | 150 |  | 20 |  | 40 | **0.479** | *0.34* | **0.946** | *0.48* | **0.566** | *0.76* | **0.056** | *0.80* | **0.512** | *0.59* |
|  |  |  | 80 |  | 300 |  | 40 |  | 80 | **0.502** | *0.43* | **0.979** | *0.61* | **0.687** | *0.61* | **0.346** | *0.50* | **0.628** | *0.54* |
|  |  |  | 160 |  | 600 |  | 80 |  | 160 | **0.561** | *0.57* | **0.997** | *0.55* | **0.793** | *0.51* | **0.698** | *0.42* | **0.762** | *0.51* |
|  |  | 500 | 10 | 500 | 37.5 | 1000 | 5 | 1250 | 10 | **0.584** | *0.34* | **0.659** | *0.63* | **0.655** | *0.76* | **0.051** | *0.71* | **0.487** | *0.61* |
|  |  |  | 20 |  | 75 |  | 10 |  | 20 | **0.580** | *0.37* | **0.787** | *0.66* | **0.786** | *0.44* | **0.060** | *0.81* | **0.553** | *0.57* |
|  |  |  | 40 |  | 150 |  | 20 |  | 40 | **0.565** | *0.43* | **0.923** | *0.64* | **0.872** | *0.27* | **0.105** | *0.86* | **0.616** | *0.55* |
|  |  |  | 80 |  | 300 |  | 40 |  | 80 | **0.593** | *0.49* | **0.977** | *0.67* | **0.927** | *0.18* | **0.294** | *0.75* | **0.698** | *0.52* |
|  |  |  | 160 |  | 600 |  | 80 |  | 160 | **0.633** | *0.61* | **0.997** | *0.55* | **0.961** | *0.12* | **0.570** | *0.69* | **0.790** | *0.49* |
|  |  | 1000 | 10 | 1000 | 37.5 | 2000 | 5 | 2500 | 10 | **0.514** | *0.81* | **0.915** | *0.45* | **0.969** | *0.12* | **0.981** | *0.36* | **0.845** | *0.44* |
|  |  |  | 20 |  | 75 |  | 10 |  | 20 | **0.518** | *0.83* | **0.946** | *0.46* | **0.975** | *0.10* | **0.976** | *0.38* | **0.854** | *0.44* |
|  |  |  | 40 |  | 150 |  | 20 |  | 40 | **0.534** | *0.84* | **0.967** | *0.54* | **0.983** | *0.08* | **0.985** | *0.36* | **0.867** | *0.45* |
|  |  |  | 80 |  | 300 |  | 40 |  | 80 | **0.561** | *0.88* | **0.984** | *0.65* | **0.991** | *0.04* | **0.988** | *0.35* | **0.881** | *0.48* |
|  |  |  | 160 |  | 600 |  | 80 |  | 160 | **0.668** | *0.79* | **0.997** | *0.59* | **0.995** | *0.03* | **0.993** | *0.34* | **0.913** | *0.44* |
| **SP2509** | **Etoposide** | 62.5 | 18.8 | 62.5 | 50 | 125 | 50 | 157 | 50 | **0.035** | *5.00* | **0.022** | *1.00* | **0.229** | *1.65* | **0.052** | *0.47* | **0.084** | *2.03* |
|  |  |  | 37.5 |  | 100 |  | 100 |  | 100 | **0.010** | *5.00* | **0.115** | *0.75* | **0.367** | *1.22* | **0.069** | *0.70* | **0.140** | *1.92* |
|  |  |  | 75 |  | 200 |  | 200 |  | 200 | **0.015** | *5.00* | **0.506** | *0.62* | **0.491** | *1.13* | **0.068** | *1.34* | **0.270** | *2.02* |
|  |  |  | 150 |  | 400 |  | 400 |  | 400 | **0.062** | *5.00* | **0.923** | *0.48* | **0.630** | *0.99* | **0.378** | *0.58* | **0.498** | *1.76* |
|  |  |  | 300 |  | 800 |  | 800 |  | 800 | **0.047** | *5.00* | **0.986** | *0.51* | **0.763** | *0.81* | **0.741** | *0.37* | **0.634** | *1.67* |
|  |  | 125 | 18.8 | 125 | 50 | 250 | 50 | 313 | 50 | **0.189** | *0.46* | **0.030** | *1.24* | **0.273** | *1.61* | **0.029** | *0.77* | **0.130** | *1.02* |
|  |  |  | 37.5 |  | 100 |  | 100 |  | 100 | **0.196** | *0.55* | **0.115** | *0.93* | **0.434** | *1.06* | **0.050** | *0.95* | **0.199** | *0.87* |
|  |  |  | 75 |  | 200 |  | 200 |  | 200 | **0.217** | *0.65* | **0.448** | *0.73* | **0.555** | *0.94* | **0.090** | *1.14* | **0.328** | *0.87* |
|  |  |  | 150 |  | 400 |  | 400 |  | 400 | **0.174** | *1.60* | **0.879** | *0.60* | **0.725** | *0.63* | **0.382** | *0.62* | **0.540** | *0.86* |
|  |  |  | 300 |  | 800 |  | 800 |  | 800 | **0.226** | *1.60* | **0.984** | *0.53* | **0.790** | *0.71* | **0.742** | *0.41* | **0.685** | *0.81* |
|  |  | 250 | 18.8 | 250 | 50 | 500 | 50 | 625 | 50 | **0.482** | *0.23* | **0.132** | *0.91* | **0.414** | *1.23* | **0.022** | *1.10* | **0.263** | *0.87* |
|  |  |  | 37.5 |  | 100 |  | 100 |  | 100 | **0.488** | *0.23* | **0.222** | *0.89* | **0.516** | *1.02* | **0.030** | *1.51* | **0.314** | *0.91* |
|  |  |  | 75 |  | 200 |  | 200 |  | 200 | **0.496** | *0.24* | **0.510** | *0.78* | **0.639** | *0.81* | **0.111** | *1.10* | **0.439** | *0.73* |
|  |  |  | 150 |  | 400 |  | 400 |  | 400 | **0.462** | *0.31* | **0.894** | *0.60* | **0.791** | *0.50* | **0.462** | *0.59* | **0.652** | *0.50* |
|  |  |  | 300 |  | 800 |  | 800 |  | 800 | **0.503** | *0.32* | **0.984** | *0.55* | **0.813** | *0.68* | **0.718** | *0.52* | **0.754** | *0.51* |
|  |  | 500 | 18.8 | 500 | 50 | 1000 | 50 | 1250 | 50 | **0.614** | *0.29* | **0.491** | *0.60* | **0.684** | *0.72* | **0.099** | *0.74* | **0.472** | *0.59* |
|  |  |  | 37.5 |  | 100 |  | 100 |  | 100 | **0.609** | *0.30* | **0.557** | *0.66* | **0.816** | *0.40* | **0.084** | *1.06* | **0.516** | *0.60* |
|  |  |  | 75 |  | 200 |  | 200 |  | 200 | **0.611** | *0.30* | **0.677** | *0.74* | **0.876** | *0.29* | **0.212** | *0.92* | **0.594** | *0.56* |
|  |  |  | 150 |  | 400 |  | 400 |  | 400 | **0.614** | *0.31* | **0.898** | *0.66* | **0.914** | *0.23* | **0.365** | *0.94* | **0.698** | *0.53* |
|  |  |  | 300 |  | 800 |  | 800 |  | 800 | **0.616** | *0.33* | **0.974** | *0.69* | **0.925** | *0.26* | **0.684** | *0.72* | **0.800** | *0.50* |
|  |  | 1000 | 18.8 | 1000 | 50 | 2000 | 50 | 2500 | 50 | **0.558** | *0.69* | **0.764** | *0.57* | **0.973** | *0.11* | **0.985** | *0.35* | **0.820** | *0.43* |
|  |  |  | 37.5 |  | 100 |  | 100 |  | 100 | **0.552** | *0.70* | **0.804** | *0.59* | **0.979** | *0.09* | **0.977** | *0.38* | **0.828** | *0.44* |
|  |  |  | 75 |  | 200 |  | 200 |  | 200 | **0.564** | *0.69* | **0.807** | *0.75* | **0.982** | *0.08* | **0.987** | *0.34* | **0.835** | *0.46* |
|  |  |  | 150 |  | 400 |  | 400 |  | 400 | **0.557** | *0.72* | **0.913** | *0.74* | **0.986** | *0.06* | **0.987** | *0.35* | **0.861** | *0.47* |
|  |  |  | 300 |  | 800 |  | 800 |  | 800 | **0.569** | *0.73* | **0.964** | *0.85* | **0.989** | *0.06* | **0.991** | *0.34* | **0.878** | *0.49* |
| **SP2509** | **Romidepsin** | 62.5 | 0.25 | 62.5 | 0.25 | 125 | 0.25 | 157 | 0.25 | **0.039** | *1.25* | **-0.001** | *5.00* | **0.032** | *3.72* | **0.031** | *0.22* | **0.025** | *2.55* |
|  |  |  | 0.5 |  | 0.5 |  | 0.5 |  | 0.5 | **0.055** | *1.47* | **-0.009** | *5.00* | **0.044** | *3.21* | **0.044** | *0.32* | **0.034** | *2.50* |
|  |  |  | 1 |  | 1 |  | 1 |  | 1 | **0.221** | *0.96* | **0.381** | *0.68* | **0.051** | *3.65* | **0.051** | *0.53* | **0.176** | *1.45* |
|  |  |  | 2 |  | 2 |  | 2 |  | 2 | **0.878** | *0.28* | **0.996** | *0.39* | **0.582** | *0.99* | **0.175** | *0.59* | **0.658** | *0.56* |
|  |  |  | 4 |  | 4 |  | 4 |  | 4 | **0.879** | *0.55* | **0.997** | *0.74* | **0.878** | *0.95* | **0.598** | *0.52* | **0.838** | *0.69* |
|  |  | 125 | 0.25 | 125 | 0.25 | 250 | 0.25 | 313 | 0.25 | **0.038** | *1.95* | **0.036** | *0.95* | **0.072** | *3.36* | **0.022** | *0.33* | **0.042** | *1.65* |
|  |  |  | 0.5 |  | 0.5 |  | 0.5 |  | 0.5 | **0.087** | *1.41* | **0.017** | *1.64* | **0.070** | *3.77* | **0.040** | *0.41* | **0.054** | *1.81* |
|  |  |  | 1 |  | 1 |  | 1 |  | 1 | **0.326** | *0.79* | **0.351** | *0.78* | **0.115** | *3.01* | **0.056** | *0.59* | **0.212** | *1.29* |
|  |  |  | 2 |  | 2 |  | 2 |  | 2 | **0.874** | *0.30* | **0.996** | *0.40* | **0.660** | *0.94* | **0.196** | *0.61* | **0.682** | *0.56* |
|  |  |  | 4 |  | 4 |  | 4 |  | 4 | **0.876** | *0.57* | **0.997** | *0.75* | **0.872** | *1.00* | **0.615** | *0.54* | **0.840** | *0.72* |
|  |  | 250 | 0.25 | 250 | 0.25 | 500 | 0.25 | 625 | 0.25 | **0.413** | *0.40* | **0.134** | *0.82* | **0.295** | *1.54* | **0.030** | *0.46* | **0.218** | *0.80* |
|  |  |  | 0.5 |  | 0.5 |  | 0.5 |  | 0.5 | **0.407** | *0.53* | **0.120** | *1.07* | **0.283** | *1.79* | **0.025** | *0.64* | **0.209** | *1.01* |
|  |  |  | 1 |  | 1 |  | 1 |  | 1 | **0.614** | *0.46* | **0.476** | *0.80* | **0.366** | *1.64* | **0.046** | *0.78* | **0.376** | *0.92* |
|  |  |  | 2 |  | 2 |  | 2 |  | 2 | **0.858** | *0.35* | **0.997** | *0.39* | **0.790** | *0.79* | **0.237** | *0.67* | **0.720** | *0.55* |
|  |  |  | 4 |  | 4 |  | 4 |  | 4 | **0.876** | *0.59* | **0.998** | *0.71* | **0.862** | *1.10* | **0.669** | *0.58* | **0.851** | *0.74* |
|  |  | 500 | 0.25 | 500 | 0.25 | 1000 | 0.25 | 1250 | 0.25 | **0.599** | *0.38* | **0.484** | *0.61* | **0.534** | *1.24* | **0.033** | *0.76* | **0.412** | *0.75* |
|  |  |  | 0.5 |  | 0.5 |  | 0.5 |  | 0.5 | **0.759** | *0.27* | **0.474** | *0.76* | **0.586** | *1.14* | **0.041** | *0.85* | **0.465** | *0.76* |
|  |  |  | 1 |  | 1 |  | 1 |  | 1 | **0.804** | *0.32* | **0.704** | *0.75* | **0.749** | *0.80* | **0.074** | *0.93* | **0.583** | *0.70* |
|  |  |  | 2 |  | 2 |  | 2 |  | 2 | **0.852** | *0.41* | **0.997** | *0.39* | **0.925** | *0.51* | **0.298** | *0.80* | **0.768** | *0.53* |
|  |  |  | 4 |  | 4 |  | 4 |  | 4 | **0.879** | *0.63* | **0.998** | *0.70* | **0.953** | *0.70* | **0.812** | *0.58* | **0.910** | *0.65* |
|  |  | 1000 | 0.25 | 1000 | 0.25 | 2000 | 0.25 | 2500 | 0.25 | **0.527** | *0.85* | **0.812** | *0.51* | **0.967** | *0.16* | **0.967** | *0.41* | **0.818** | *0.48* |
|  |  |  | 0.5 |  | 0.5 |  | 0.5 |  | 0.5 | **0.697** | *0.55* | **0.909** | *0.44* | **0.972** | *0.17* | **0.978** | *0.39* | **0.889** | *0.39* |
|  |  |  | 1 |  | 1 |  | 1 |  | 1 | **0.860** | *0.34* | **0.991** | *0.30* | **0.988** | *0.14* | **0.946** | *0.48* | **0.946** | *0.32* |
|  |  |  | 2 |  | 2 |  | 2 |  | 2 | **0.946** | *0.25* | **0.998** | *0.38* | **0.997** | *0.11* | **0.971** | *0.45* | **0.978** | *0.30* |
|  |  |  | 4 |  | 4 |  | 4 |  | 4 | **0.978** | *0.24* | **0.999** | *0.67* | **0.999** | *0.17* | **0.994** | *0.37* | **0.992** | *0.36* |
| **SP2509** | **SN-38** | 62.5 | 0.0625 | 62.5 | 0.047 | 125 | 0.047 | 157 | 0.047 | **-0.088** | *5.00* | **0.018** | *0.60* | **0.042** | *3.24* | **0.009** | *0.25* | **-0.005** | *2.27* |
|  |  |  | 0.125 |  | 0.094 |  | 0.094 |  | 0.094 | **-0.054** | *5.00* | **0.003** | *1.67* | **0.086** | *2.18* | **0.017** | *0.34* | **0.013** | *2.30* |
|  |  |  | 0.25 |  | 0.188 |  | 0.188 |  | 0.188 | **-0.069** | *5.00* | **0.013** | *1.03* | **0.187** | *1.60* | **0.035** | *0.48* | **0.041** | *2.03* |
|  |  |  | 0.5 |  | 0.375 |  | 0.375 |  | 0.375 | **-0.018** | *5.00* | **0.083** | *0.74* | **0.402** | *1.26* | **0.222** | *0.50* | **0.172** | *1.87* |
|  |  |  | 1 |  | 0.75 |  | 0.75 |  | 0.75 | **0.127** | *0.66* | **0.619** | *0.61* | **0.853** | *0.57* | **0.713** | *0.51* | **0.578** | *0.59* |
|  |  | 125 | 0.0625 | 125 | 0.047 | 250 | 0.047 | 313 | 0.047 | **0.081** | *0.74* | **0.032** | *0.82* | **0.090** | *2.89* | **-0.001** | *5.00* | **0.050** | *2.36* |
|  |  |  | 0.125 |  | 0.094 |  | 0.094 |  | 0.094 | **0.022** | *2.17* | **0.026** | *0.99* | **0.150** | *2.06* | **0.013** | *0.45* | **0.053** | *1.42* |
|  |  |  | 0.25 |  | 0.188 |  | 0.188 |  | 0.188 | **0.041** | *1.41* | **0.015** | *1.52* | **0.273** | *1.50* | **0.008** | *0.84* | **0.084** | *1.32* |
|  |  |  | 0.5 |  | 0.375 |  | 0.375 |  | 0.375 | **0.102** | *0.81* | **0.105** | *0.87* | **0.606** | *0.83* | **0.228** | *0.55* | **0.260** | *0.76* |
|  |  |  | 1 |  | 0.75 |  | 0.75 |  | 0.75 | **0.346** | *0.47* | **0.554** | *0.70* | **0.873** | *0.54* | **0.748** | *0.52* | **0.630** | *0.56* |
|  |  | 250 | 0.0625 | 250 | 0.047 | 500 | 0.047 | 625 | 0.047 | **0.412** | *0.29* | **0.149** | *0.64* | **0.334** | *1.32* | **0.015** | *0.49* | **0.228** | *0.69* |
|  |  |  | 0.125 |  | 0.094 |  | 0.094 |  | 0.094 | **0.433** | *0.29* | **0.146** | *0.71* | **0.416** | *1.10* | **0.004** | *0.81* | **0.250** | *0.73* |
|  |  |  | 0.25 |  | 0.188 |  | 0.188 |  | 0.188 | **0.434** | *0.32* | **0.185** | *0.73* | **0.593** | *0.77* | **0.014** | *0.89* | **0.307** | *0.68* |
|  |  |  | 0.5 |  | 0.375 |  | 0.375 |  | 0.375 | **0.458** | *0.37* | **0.255** | *0.81* | **0.793** | *0.54* | **0.332** | *0.57* | **0.460** | *0.57* |
|  |  |  | 1 |  | 0.75 |  | 0.75 |  | 0.75 | **0.601** | *0.37* | **0.679** | *0.69* | **0.866** | *0.62* | **0.800** | *0.55* | **0.736** | *0.56* |
|  |  | 500 | 0.0625 | 500 | 0.047 | 1000 | 0.047 | 1250 | 0.047 | **0.601** | *0.31* | **0.494** | *0.50* | **0.597** | *0.97* | **0.021** | *0.79* | **0.428** | *0.64* |
|  |  |  | 0.125 |  | 0.094 |  | 0.094 |  | 0.094 | **0.594** | *0.33* | **0.530** | *0.50* | **0.597** | *1.05* | **0.058** | *0.74* | **0.445** | *0.65* |
|  |  |  | 0.25 |  | 0.188 |  | 0.188 |  | 0.188 | **0.635** | *0.32* | **0.590** | *0.52* | **0.652** | *1.00* | **0.097** | *0.81* | **0.494** | *0.66* |
|  |  |  | 0.5 |  | 0.375 |  | 0.375 |  | 0.375 | **0.689** | *0.32* | **0.661** | *0.59* | **0.774** | *0.80* | **0.433** | *0.70* | **0.639** | *0.60* |
|  |  |  | 1 |  | 0.75 |  | 0.75 |  | 0.75 | **0.776** | *0.33* | **0.842** | *0.62* | **0.842** | *0.85* | **0.813** | *0.68* | **0.818** | *0.62* |
|  |  | 1000 | 0.0625 | 1000 | 0.047 | 2000 | 0.047 | 2500 | 0.047 | **0.588** | *0.53* | **0.732** | *0.55* | **0.963** | *0.15* | **0.970** | *0.41* | **0.813** | *0.41* |
|  |  |  | 0.125 |  | 0.094 |  | 0.094 |  | 0.094 | **0.600** | *0.52* | **0.730** | *0.59* | **0.963** | *0.17* | **0.976** | *0.40* | **0.817** | *0.42* |
|  |  |  | 0.25 |  | 0.188 |  | 0.188 |  | 0.188 | **0.614** | *0.53* | **0.740** | *0.64* | **0.964** | *0.19* | **0.980** | *0.42* | **0.824** | *0.44* |
|  |  |  | 0.5 |  | 0.375 |  | 0.375 |  | 0.375 | **0.628** | *0.56* | **0.768** | *0.71* | **0.968** | *0.22* | **0.985** | *0.44* | **0.837** | *0.48* |
|  |  |  | 1 |  | 0.75 |  | 0.75 |  | 0.75 | **0.699** | *0.54* | **0.821** | *0.85* | **0.975** | *0.26* | **0.989** | *0.50* | **0.871** | *0.54* |
| **SP2509** | **Vincristine** | 62.5 | 0.625 | 62.5 | 0.0375 | 125 | 0.125 | 157 | 0.188 | **0.926** | *0.53* | **0.043** | *0.38* | **0.026** | *5.00* | **0.035** | *0.85* | **0.257** | *1.69* |
|  |  |  | 1.25 |  | 0.075 |  | 0.25 |  | 0.375 | **0.954** | *0.86* | **0.022** | *0.62* | **0.036** | *5.00* | **0.167** | *0.75* | **0.295** | *1.81* |
|  |  |  | 2.5 |  | 0.15 |  | 0.5 |  | 0.75 | **0.954** | *1.71* | **0.029** | *0.70* | **0.243** | *2.23* | **0.669** | *0.51* | **0.474** | *1.29* |
|  |  |  | 5 |  | 0.3 |  | 1 |  | 1.5 | **0.942** | *3.81* | **0.064** | *0.81* | **0.458** | *1.17* | **0.958** | *0.34* | **0.605** | *1.53* |
|  |  |  | 10 |  | 0.6 |  | 2 |  | 3 | **0.932** | *5.00* | **0.824** | *0.53* | **0.634** | *0.86* | **0.989** | *0.35* | **0.844** | *1.69* |
|  |  | 125 | 0.625 | 125 | 0.0375 | 250 | 0.125 | 313 | 0.188 | **0.917** | *0.57* | **0.056** | *0.60* | **0.062** | *5.00* | **0.028** | *1.00* | **0.266** | *1.79* |
|  |  |  | 1.25 |  | 0.075 |  | 0.25 |  | 0.375 | **0.948** | *0.91* | **0.044** | *0.75* | **0.088** | *5.00* | **0.195** | *0.74* | **0.319** | *1.85* |
|  |  |  | 2.5 |  | 0.15 |  | 0.5 |  | 0.75 | **0.949** | *1.80* | **0.033** | *1.02* | **0.329** | *1.61* | **0.693** | *0.53* | **0.501** | *1.24* |
|  |  |  | 5 |  | 0.3 |  | 1 |  | 1.5 | **0.931** | *4.10* | **0.111** | *0.88* | **0.511** | *1.05* | **0.958** | *0.36* | **0.628** | *1.60* |
|  |  |  | 10 |  | 0.6 |  | 2 |  | 3 | **0.921** | *5.00* | **0.857** | *0.53* | **0.650** | *0.88* | **0.980** | *0.47* | **0.852** | *1.72* |
|  |  | 250 | 0.625 | 250 | 0.0375 | 500 | 0.125 | 625 | 0.188 | **0.914** | *0.60* | **0.195** | *0.61* | **0.255** | *2.04* | **0.019** | *1.37* | **0.346** | *1.16* |
|  |  |  | 1.25 |  | 0.075 |  | 0.25 |  | 0.375 | **0.939** | *1.00* | **0.208** | *0.64* | **0.247** | *2.57* | **0.202** | *0.84* | **0.399** | *1.26* |
|  |  |  | 2.5 |  | 0.15 |  | 0.5 |  | 0.75 | **0.939** | *1.95* | **0.211** | *0.74* | **0.385** | *1.69* | **0.712** | *0.58* | **0.562** | *1.24* |
|  |  |  | 5 |  | 0.3 |  | 1 |  | 1.5 | **0.932** | *4.10* | **0.413** | *0.68* | **0.597** | *0.92* | **0.928** | *0.52* | **0.718** | *1.55* |
|  |  |  | 10 |  | 0.6 |  | 2 |  | 3 | **0.916** | *5.00* | **0.885** | *0.55* | **0.721** | *0.72* | **0.967** | *0.65* | **0.872** | *1.73* |
|  |  | 500 | 0.625 | 500 | 0.0375 | 1000 | 0.125 | 1250 | 0.188 | **0.897** | *0.69* | **0.532** | *0.55* | **0.515** | *1.29* | **0.207** | *0.75* | **0.538** | *0.82* |
|  |  |  | 1.25 |  | 0.075 |  | 0.25 |  | 0.375 | **0.917** | *1.17* | **0.530** | *0.59* | **0.553** | *1.20* | **0.597** | *0.60* | **0.649** | *0.89* |
|  |  |  | 2.5 |  | 0.15 |  | 0.5 |  | 0.75 | **0.912** | *2.35* | **0.489** | *0.72* | **0.693** | *0.75* | **0.808** | *0.61* | **0.725** | *1.11* |
|  |  |  | 5 |  | 0.3 |  | 1 |  | 1.5 | **0.891** | *5.00* | **0.631** | *0.71* | **0.829** | *0.41* | **0.915** | *0.67* | **0.816** | *1.70* |
|  |  |  | 10 |  | 0.6 |  | 2 |  | 3 | **0.887** | *5.00* | **0.803** | *0.77* | **0.872** | *0.36* | **0.957** | *0.84* | **0.880** | *1.74* |
|  |  | 1000 | 0.625 | 1000 | 0.0375 | 2000 | 0.125 | 2500 | 0.188 | **0.809** | *1.10* | **0.855** | *0.46* | **0.957** | *0.17* | **0.981** | *0.39* | **0.900** | *0.53* |
|  |  |  | 1.25 |  | 0.075 |  | 0.25 |  | 0.375 | **0.818** | *1.88* | **0.833** | *0.53* | **0.960** | *0.16* | **0.983** | *0.41* | **0.898** | *0.74* |
|  |  |  | 2.5 |  | 0.15 |  | 0.5 |  | 0.75 | **0.826** | *3.43* | **0.859** | *0.54* | **0.960** | *0.16* | **0.985** | *0.44* | **0.907** | *1.14* |
|  |  |  | 5 |  | 0.3 |  | 1 |  | 1.5 | **0.822** | *5.00* | **0.881** | *0.61* | **0.964** | *0.15* | **0.984** | *0.55* | **0.913** | *1.58* |
|  |  |  | 10 |  | 0.6 |  | 2 |  | 3 | **0.817** | *5.00* | **0.925** | *0.71* | **0.967** | *0.15* | **0.989** | *0.66* | **0.924** | *1.63* |
| **Vincristine** | **Doxorubicin** | 0.125 | 10 | 0.0375 | 37.5 | 0.125 | 5 | 0.188 | 10 | **0.934** | *0.04* | **-0.006** | *5.00* | **0.130** | *1.91* | **0.024** | *1.12* | **0.271** | *2.02* |
|  |  |  | 20 |  | 75 |  | 10 |  | 20 | **0.931** | *0.05* | **-0.001** | *5.00* | **0.181** | *1.62* | **0.015** | *1.67* | **0.281** | *2.09* |
|  |  |  | 40 |  | 150 |  | 20 |  | 40 | **0.934** | *0.06* | **0.356** | *1.65* | **0.293** | *1.22* | **0.013** | *2.41* | **0.399** | *1.33* |
|  |  |  | 80 |  | 300 |  | 40 |  | 80 | **0.918** | *0.08* | **0.915** | *1.04* | **0.418** | *1.15* | **0.053** | *1.69* | **0.576** | *0.99* |
|  |  |  | 160 |  | 600 |  | 80 |  | 160 | **0.508** | *0.20* | **0.984** | *1.06* | **0.519** | *1.36* | **-0.004** | *5.00* | **0.502** | *1.91* |
|  |  | 0.25 | 10 | 0.075 | 37.5 | 0.25 | 5 | 0.375 | 10 | **0.951** | *0.08* | **-0.001** | *5.00* | **0.171** | *2.10* | **0.120** | *0.91* | **0.310** | *2.02* |
|  |  |  | 20 |  | 75 |  | 10 |  | 20 | **0.945** | *0.08* | **0.018** | *3.15* | **0.210** | *1.88* | **0.083** | *1.20* | **0.314** | *1.58* |
|  |  |  | 40 |  | 150 |  | 20 |  | 40 | **0.948** | *0.09* | **0.363** | *1.67* | **0.298** | *1.50* | **0.082** | *1.41* | **0.423** | *1.17* |
|  |  |  | 80 |  | 300 |  | 40 |  | 80 | **0.942** | *0.12* | **0.917** | *1.06* | **0.426** | *1.25* | **0.056** | *2.25* | **0.585** | *1.17* |
|  |  |  | 160 |  | 600 |  | 80 |  | 160 | **0.544** | *0.28* | **0.983** | *1.09* | **0.527** | *1.41* | **0.036** | *4.17* | **0.523** | *1.74* |
|  |  | 0.5 | 10 | 0.15 | 37.5 | 0.5 | 5 | 0.75 | 10 | **0.960** | *0.14* | **-0.002** | *5.00* | **0.342** | *1.12* | **0.607** | *0.56* | **0.477** | *1.70* |
|  |  |  | 20 |  | 75 |  | 10 |  | 20 | **0.958** | *0.15* | **0.027** | *2.87* | **0.345** | *1.28* | **0.572** | *0.62* | **0.475** | *1.23* |
|  |  |  | 40 |  | 150 |  | 20 |  | 40 | **0.957** | *0.16* | **0.321** | *1.89* | **0.388** | *1.29* | **0.521** | *0.73* | **0.547** | *1.02* |
|  |  |  | 80 |  | 300 |  | 40 |  | 80 | **0.944** | *0.19* | **0.885** | *1.27* | **0.464** | *1.29* | **0.397** | *1.06* | **0.672** | *0.95* |
|  |  |  | 160 |  | 600 |  | 80 |  | 160 | **0.609** | *0.43* | **0.984** | *1.12* | **0.548** | *1.43* | **0.306** | *1.62* | **0.612** | *1.15* |
|  |  | 1 | 10 | 0.3 | 37.5 | 1 | 5 | 1.5 | 10 | **0.960** | *0.27* | **0.027** | *1.91* | **0.503** | *0.87* | **0.964** | *0.29* | **0.614** | *0.83* |
|  |  |  | 20 |  | 75 |  | 10 |  | 20 | **0.963** | *0.27* | **0.058** | *2.43* | **0.503** | *0.95* | **0.963** | *0.30* | **0.622** | *0.99* |
|  |  |  | 40 |  | 150 |  | 20 |  | 40 | **0.960** | *0.29* | **0.311** | *2.12* | **0.526** | *1.00* | **0.960** | *0.32* | **0.689** | *0.93* |
|  |  |  | 80 |  | 300 |  | 40 |  | 80 | **0.955** | *0.32* | **0.882** | *1.39* | **0.556** | *1.13* | **0.933** | *0.44* | **0.831** | *0.82* |
|  |  |  | 160 |  | 600 |  | 80 |  | 160 | **0.739** | *0.63* | **0.983** | *1.21* | **0.589** | *1.43* | **0.837** | *0.78* | **0.787** | *1.01* |
|  |  | 2 | 10 | 0.6 | 37.5 | 2 | 5 | 3 | 10 | **0.964** | *0.52* | **0.356** | *1.14* | **0.674** | *0.64* | **0.993** | *0.26* | **0.747** | *0.64* |
|  |  |  | 20 |  | 75 |  | 10 |  | 20 | **0.961** | *0.54* | **0.368** | *1.51* | **0.681** | *0.66* | **0.991** | *0.30* | **0.750** | *0.75* |
|  |  |  | 40 |  | 150 |  | 20 |  | 40 | **0.965** | *0.53* | **0.528** | *1.86* | **0.694** | *0.68* | **0.991** | *0.30* | **0.795** | *0.84* |
|  |  |  | 80 |  | 300 |  | 40 |  | 80 | **0.958** | *0.58* | **0.888** | *1.59* | **0.718** | *0.71* | **0.988** | *0.35* | **0.888** | *0.81* |
|  |  |  | 160 |  | 600 |  | 80 |  | 160 | **0.870** | *0.89* | **0.981** | *1.42* | **0.672** | *1.25* | **0.982** | *0.46* | **0.876** | *1.00* |
